# HIV-attributable causes of death in the medical ward at the Chris Hani Baragwanath Hospital, South Africa

**DOI:** 10.1371/journal.pone.0215591

**Published:** 2019-05-06

**Authors:** Andrew Black, Freddy Sitas, Trust Chibrawara, Zoe Gill, Mmamapudi Kubanje, Brian Williams

**Affiliations:** 1 Department of Internal Medicine, University of the Witwatersrand, Johannesburg, South Africa; 2 Centre for Primary Health Care and Equity, School of Public Health and Community Medicine, University of New South Wales, Kensington, Australia; 3 Menzies Centre for Health Policy, Sydney School of Public Health, University of Sydney, Camperdown, Australia; 4 South African Centre for Epidemiological Modelling and Analysis, Stellenbosch University, Stellenbosch, South Africa; University of KwaZulu-Natal, SOUTH AFRICA

## Abstract

**Introduction:**

Data on the association between HIV infection and deaths from underlying medical conditions are needed to understand and assess the impact of HIV on mortality. We present data on mortality in the Chris Hani Baragwanath Hospital (CHBH) South Africa and analyse the relationship between each cause of death and HIV.

**Methods:**

From 2006 to 2009 data were collected on 15,725 deaths including age, sex, day of admittance and of death, HIV status, ART initiation and CD4+ cell counts. Causes of death associated with HIV were cases, causes of death not associated with HIV were controls. We calculate the odds-ratios (ORs) for being HIV-positive and for each AIDS related condition the disease-attributable fraction (DAF) and the population-attributable fraction (PAF) due to HIV for cases relative to controls.

**Results:**

Among those that died, the prevalence of HIV was 61% and of acquired immune deficiency syndrome (AIDS) related conditions was 69%. The HIV-attributable fraction was 36% in the whole sample and 60% in those that were HIV-positive. Cryptococcosis, Kaposi’s sarcoma and *Pneumocystis jirovecii*, TB, gastroenteritis and anaemia were highly predictive of HIV with odds ratios for being HIV-positive ranging from 8 to 124, while genito-urinary conditions, meningitis, other respiratory conditions and sepsis, lymphoma and conditions of skin and bone were significantly associated with HIV with odds ratios for being HIV-positive ranging from 3 to 8. Most of the deaths attributable to HIV were among those dying of TB or of other respiratory conditions.

**Conclusions:**

The high prevalence of HIV among those that died, peaking at 70% in those aged 30 years but still 7% in those aged 80 years, demonstrates the impact of the HIV epidemic on adult mortality and on hospital services and the extent to which early anti-retroviral treatment would have reduced the burden of both. These data make it possible to better assess mortality and morbidity due to HIV in this still high prevalence setting and, in particular, to identify those causes of death that are most strongly associated with HIV.

## Introduction

A study using data collected between 2002 and 2006, immediately before these data were collected, suggested that 1.8% of deaths in a public hospital in the Eastern Cape, South Africa were due to HIV [1] but the HIV status of those that died was not recorded. A more recent study [2] suggested that in 2006 283 thousand deaths or 42% of all deaths in South Africa were attributable to HIV and the authors compared this to other estimates for 2006 of 225 thousand using the Actuarial Society of South Africa Model [3], 250 thousand using the Thembisa model [2], 270 thousand in the Global Burden of Disease study [4,5], 350 thousand using the UNAIDS Spectrum model and 354 thousand based on changes in the age-distribution of deaths over time [6]. The considerable variation in these estimates was attributed to misclassification of AIDS deaths associated with different underlying conditions [2].

The difficulty in estimating AIDS deaths is partly due to the difficulty in deciding what proportion of deaths from a particular cause, such as TB, should be attributed to HIV [7]. The published models [2–8] all agree that the number of AIDS deaths peaked in 2006 but the model estimates of mortality vary widely because the models differ in their assumptions about survival after infection without ART. Without a direct estimate of AIDS-related deaths or of survival after infection without ART, models fitted to trend data in HIV prevalence and ART coverage cannot be used to accurately determine the overall mortality attributable to HIV.

The early response of the South African Government to the epidemic of HIV was one of confused denial [9] and although the epidemic started later in South Africa than in many neighbouring countries [10,11] the provision of ART in the public health system only began in earnest in 2006 by which time AIDS deaths had reached their peak [10]. The stigma associated with HIV and the unwillingness to acknowledge the magnitude of the epidemic meant that HIV and AIDS were often omitted from death certificates with other causes of death, such as TB, listed as the preferred alternative.

Between 2006 and 2009, when these data were collected, the Chris Hani Baragwanath Hospital (CHBH) was one of the main referral hospitals in South Africa serving a population of 1.3 million or 2.4% of the population of South Africa. At that time about 300 thousand people were dying of AIDS each year [2] so that about seven thousand would have died each year in Soweto. In the CHBH there were 3.9 thousand deaths each year, on average, suggesting that more than half of all those that died of AIDS in Soweto died in the medical wards of the CHBH. In an earlier study among children in the CHBH [12] the proportion of deaths accounted for by HIV infection increased from 7% in 1992 to 46% in 1996 with an overall odds ratio of 2.85.

Here we use a detailed and extensive set of data from the CHBH where the cause of death as well as the HIV status of those that died was established in order to determine the proportion of deaths from a range of causes that are attributable to HIV before ART became widely available in the public health sector.

## Methods

### Study design

The study was conducted in the medical ward of the CHBH. The trauma, emergency, obstetrics, gynaecology, surgery, and paediatric wards were not included. For all 15,725 adults that died between January 2006 and December 2009 the hospital number, age, sex, cause of death, date of admission, date of death, HIV status, CD4+ cell count, and ART status were recorded on the Baragwanath Mortality Record (BMR). Owing to the stigma associated with HIV, clinicians were reluctant to include HIV status or AIDS defining causes of death on the official death notification forms. The BMR was developed to allow for a more accurate and detailed record of the causes of death. Attending medical consultants ascertained the underlying causes of death by reviewing the patient medical notes and completed the BMR at the time of signing the deceased’s official death certificate. Data from the BMR were entered into an Excel spread-sheet and the cause of death was classified according to the International Classification of Diseases (ICD) 10 by a trained data capturer. In cases where the code allocation was unclear to the data capturer, AB reviewed the available clinical data and assigned the ICD 10 code. Cases were excluded if fewer than 20 people were assigned to a particular cause of death (527 cases) of if a cause of death was not recorded (529 cases).

### Data cleaning

The data are missing 9 hospital numbers, 109 dates of admission, 1 date of death, no ages or sex and 529 ICD codes making up 3.4% of the sample (S1 Text, Appendices 1 and 2). In the whole sample the HIV status of 11% was given as ‘suspected’ and of 15% as ‘unknown’.

### Grouping causes of death

There were 323 individual ICD codes in the data and the number of deaths, HIV-status, mean age, number of men and women, and the groups to which they were assigned are given in the Supporting Information (S1 Text, Appendix 2). For 529 deaths (3.4% of the total sample) an ICD code was not assigned and there was uncertainty as to whether or not 527 poorly defined deaths (3.4% of the sample) were from medical conditions associated with HIV. The remaining 14,669 deaths were assigned to one of 15 medical conditions associated with HIV/AIDS or to a control group.

We carried out a case control study to determine the HIV-attributable fraction in cases compared to controls. Cases were those that died from AIDS related causes of death (with their ICD 10 codes): infectious gastroenteritis (A09), tuberculosis of the lung (A15 and A16), extra-pulmonary tuberculosis (A17 to A19 and A31), certain infectious and parasitic diseases (A41), HIV (B21 to B24), cryptococcosis (B45), pneumocystis (B59), Kaposi’s sarcoma (C46), Hodgkin’s and non-Hodgkin’s lymphoma (C82 and C85), diseases of the blood and blood forming organisms (most of D51 to D89), meningitis (G00, G03, G04, G06 and G08), pneumonia, chronic obstructive pulmonary disease, and lower respiratory tract infections (included in J01–J99), diseases of the digestive system (K72–K74), diseases of skin and bone (included in L03–L95 and M01–M99) and genitourinary conditions (N00–N39). Controls were medical conditions that are not, or are not known to be, associated with HIV and included: malignant neoplasms excluding Kaposi’s sarcoma, Hodgkin’s and non-Hodgkin’s lymphomas, disorders involving immune mechanisms or the nervous system, heart disease and stroke, and external causes of death such as injury or poisoning (S1 Text, Appendix 2).

### Statistical analysis

We first consider the overall distribution by age and gender of the people that died by fitting the number of deaths to skew-normal distributions (S1 Text, Appendix 3). Because some deaths were given a ‘suspected’ or ‘unknown’ HIV status, we use the fitted curves to estimate the proportion of those whose HIV status was ‘suspected’ or ‘unknown’ that were in fact HIV-positive or HIV-negative and use this to justify assigning HIV-suspected cases to the HIV-positive category and HIV-unknown cases to the HIV-negative category (S1 Text, Appendix 3). We then carried out a case-control analysis, adjusting for age, to determine the odds-ratio (OR) for being HIV positive for each of the AIDS related causes of death as compared to the control conditions not related to AIDS and to calculate disease and population fractions attributable to HIV (S1 Text, Appendix 4). Excluding those whose status was ‘suspected’ or ‘unknown’ did not change the overall conclusions substantially but added to the statistical uncertainty.

The odds for being HIV positive given each cause of death and the odds ratios compared to the control group were calculated in ten-year age bands. The adjusted odds ratios are the weighted averages over all ages. For four of the AIDS related causes of death, cryptococcosis, Kaposi’s sarcoma, *Pneumocystis jirovecii*, and diseases of the blood and blood forming organs, there were too few negative cases to adjust reliably for age. We therefore established a relationship between the adjusted and the crude odds ratios (S1 Text, Appendix 5) and used this to estimate the age-adjusted odds ratios for these four AIDS related causes of death. We calculate the disease attributable fraction (DAF) which is the proportion of deaths from a given disease that are attributable to HIV infection and the population attributable fraction (PAF) which is given by the DAF multiplied by the prevalence of that cause of death (S1 Text, Appendix 4).

## Results

Of the 15,981 adults that died 15,722 were included in the analysis, 7630 men and 8092 women, while 527 cases were excluded as the cause of death occurred in less than 20 patients, and an additional 529 cases were excluded as the cause of death was not recorded.

**Fig 1:**
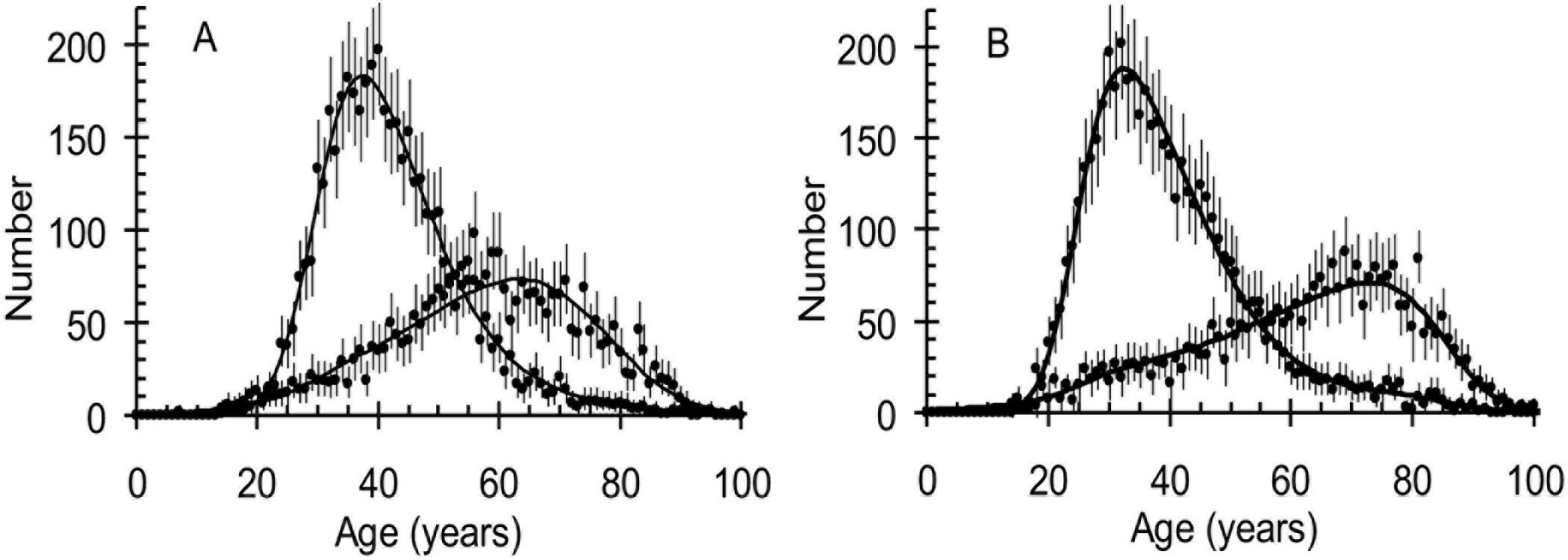
The number of A: Men and B: Women who were HIV-positive (peaks at lower ages) and who were HIV-negative (peaks at higher ages).

Of the men, 48% were HIV-positive, 15% were HIV-negative, 11% were suspected to be HIV-positive and 25% were of unknown status. The corresponding figures for women were 54%, 11%, 17% and 25%, respectively. For HIV-negative men and women the modal ages at death were 64 and 74 years, respectively; for HIV-positive men and women they were 37 and 32 years, respectively (Fig 1). CD4+ cell counts were available for 41% of those that were HIV-positive and among these the median CD4+ cell count was 45/μL (90% between 3/μL and 310/μL) showing that most of the HIV-positive patients were in late stages of HIV. Despite the availability of ART in the public sector ART coverage was low in those infected with HIV increasing from 4.9% in 2006 to 8.6% in 2009.

The distribution of time from admission to death was not significantly different for men and women or for those that were HIV-positive or HIV-negative. Amongst this deceased population, 27% had died by the day after admission and the mortality was 11% to 13% per day after that (S1 Text, Appendix 6).

### Prevalence of HIV in controls

The overall prevalence of HIV in those with the control conditions, not related to AIDS, was 27.4% and the age specific prevalence for women is given in S1 Text, Appendix 7. The peak prevalence is high at about 67% in 25–34 year olds and even in those aged 80 to 90 years the prevalence of HIV was 12% ± 3%. Comparing the prevalence of HIV in the control group to the prevalence of HIV in the ante-natal clinic surveys in the Johannesburg Municipality from 2006 to 2009 [13], where the peak prevalence was 40%, suggests an odds ratio of 3.2 but the overall distributions of deaths by age are closely matched. We discuss the this difference and the implications for this analysis in S1 Text, Appendix 7.

### Odds and odds ratios

**Fig 2:**
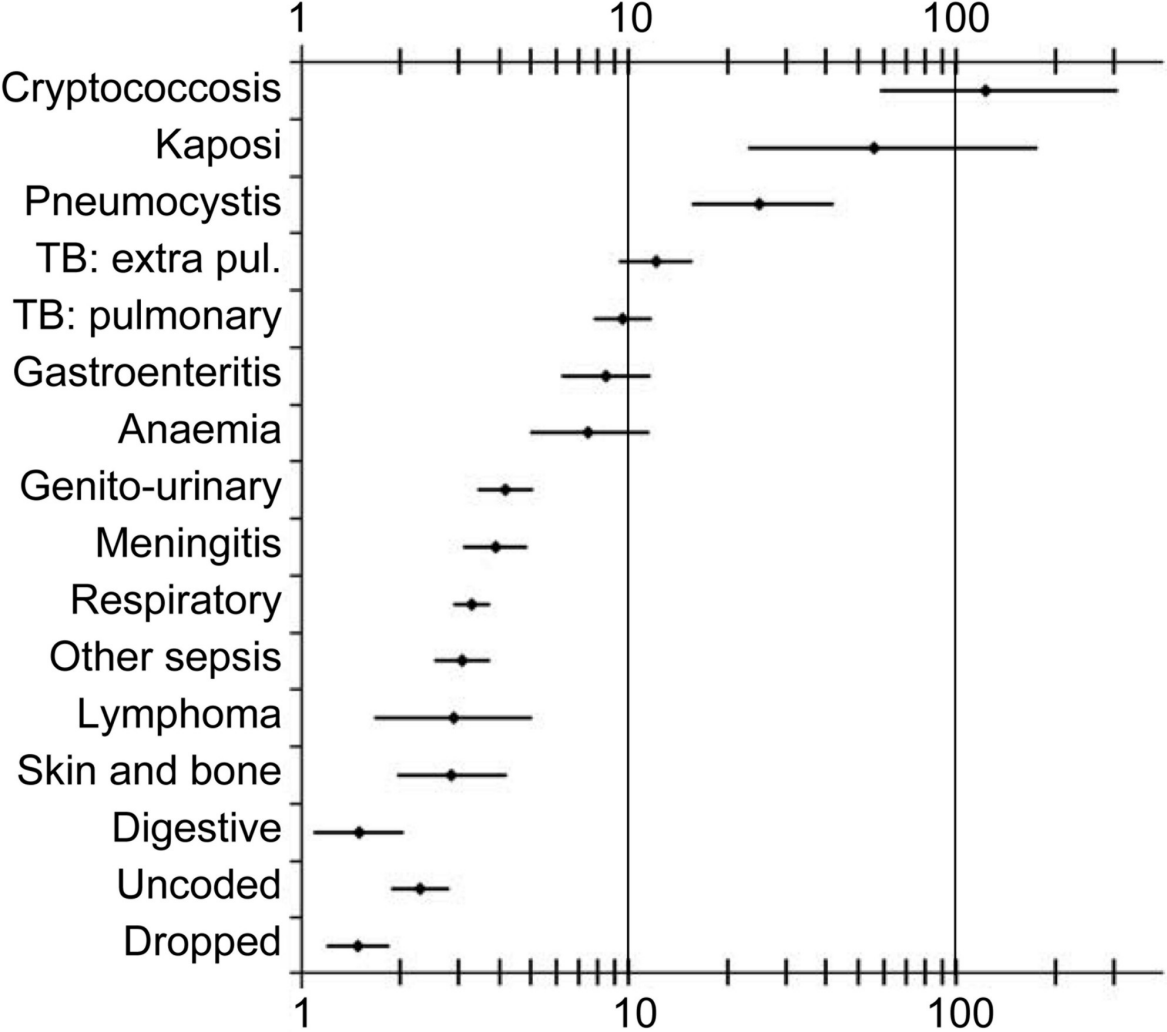
The odds ratios for being HIV-positive for particular causes of death compared to controls.

**Fig 3:**
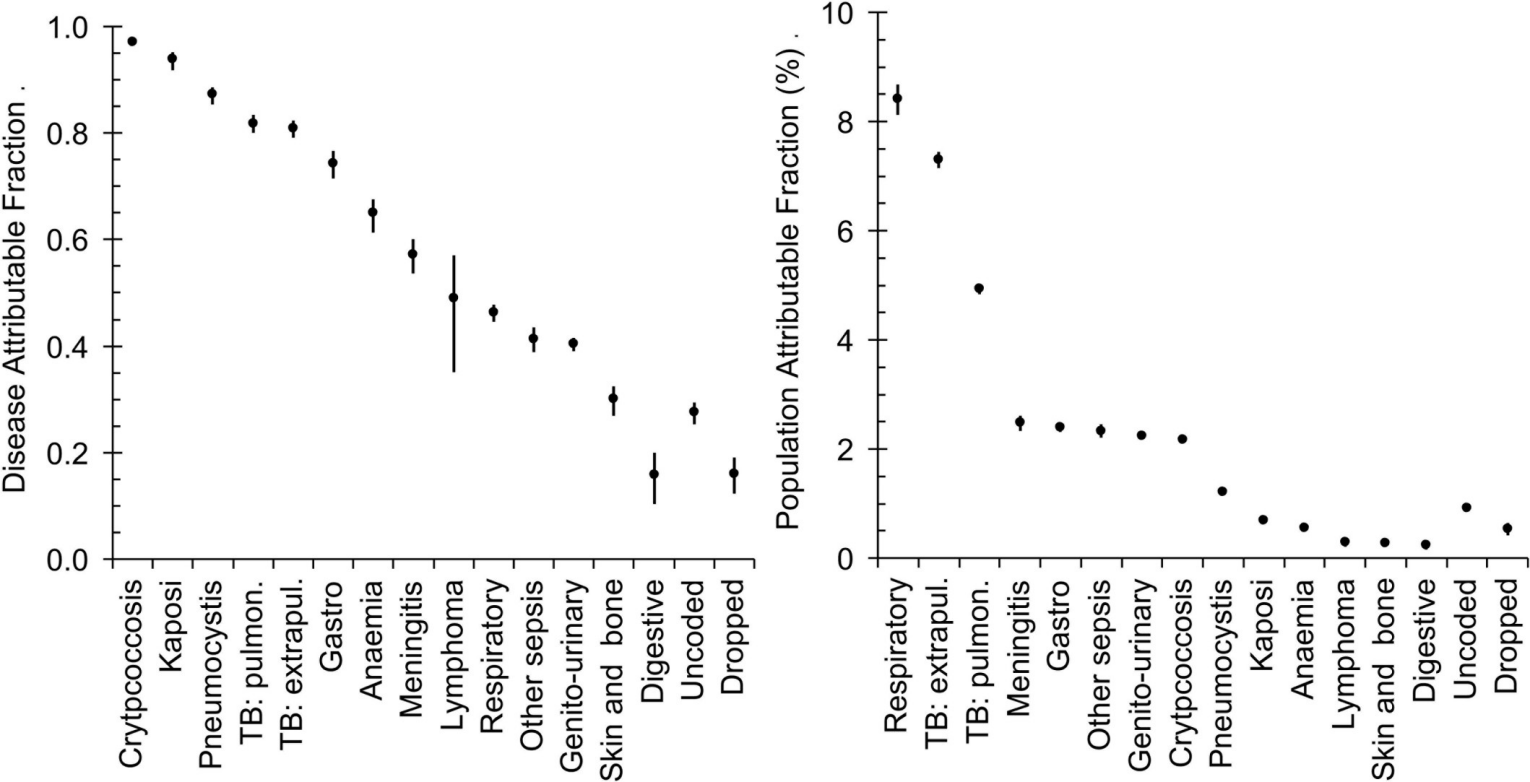
HIV disease-attributable fraction (DAF) and population-attributable fraction (PAF) for different causes of death.

The final estimates for the ORs, DAFs and PAFs for HIV are shown in Table 1 and Fig 2 and Fig 3. For cryptococcosis, Kaposi’s sarcoma, pneumocystis and tuberculosis the DAFs are range from 80% to almost 100%. For gastroenteritis, anaemia, meningitis and lymphoma the DAF range from 50% to 75%. For other respiratory conditions, sepsis, genitourinary and disorders of the skin and bone the DAF ranges from 30% to 50%. For digestive conditions the DAF is 16%.

**Table 1:** The total number, prevalence of HIV, odds ratios for HIV in those with conditions associated with HIV versus those with control conditions not associated with HIV, the disease (DAF) and population (PAF) attributable fractions for HIV. Point estimates with 95% confidence limits. (Details in S1 Text, Appendix 8).

	N	HIV+	OR	DAF (%)	PAF (%)
Cryptococcosis*	353	0.980	123.92 (59.5–310)	97.2 (96.4–97.7)	2.18 (2.16–2.19)
Kaposi*	117	0.957	56.16 (23.4–176)	94.0 (91.6–95.2)	0.70 (0.68–0.71)
Pneumocystis*	220	0.909	25.07 (15.8–41.9)	87.3 (85.2–88.7)	1.22 (1.19–1.24)
TB: pulmonary	1421	0.882	9.62 (7.93–11.7)	80.9 (78.9–82.5)	4.94 (4.82–5.04)
TB: extra-pulmon.	949	0.914	12.11 (9.46–15.5)	81.9 (79.8–83.5)	7.31 (7.13–7.45)
Gastroenteritis	509	0.843	8.53 (6.29–11.6)	74.4 (70.9–77.0)	2.41 (2.29–2.49)
Anaemia*	136	0.750	7.52 (5.06–11.4)	65.0 (60.2–68.4)	0.56 (0.52–0.59)
Meningitis	875	0.531	3.91 (3.16–4.85)	40.4 (37.9–42.6)	2.49 (2.29–2.65)
Lymphoma	684	0.769	2.91 (1.69–5.00)	57.2 (52.6–61.0)	0.30 (0.18–0.36)
Respiratory	2858	0.662	3.32 (2.95–3.74)	46.3 (43.8–48.5)	8.41 (7.96–8.82)
Sepsis	889	0.611	3.10 (2.58–3.73)	41.4 (37.4–44.7)	2.34 (2.11–2.53)
Genitourinary	95	0.747	4.18 (3.48–5.04)	49.0 (30.5–59.8)	2.25 (2.11–2.37)
Skin and bone	147	0.463	2.88 (1.98–4.18)	30.2 (22.9–35.2)	0.28 (0.21–0.33)
Digestive	240	0.479	1.50 (1.10–2.04)	15.9 (4.4–24.4)	0.24 (0.07–0.37)
Controls	4503	0.286		Reference	
Uncoded	529	0.488	2.30 (1.89–2.79)	27.6 (23.0–31.3)	0.93 (0.77–1.05)
Dropped	527	0.488	1.49 (1.20–1.84)	16.0 (8.3–22.3)	0.54 (0.28–0.75)

* Odds-ratios for these causes of death were estimated as discussed in S1 Text, Appendix 5.

HIV-attributable deaths from TB and other respiratory conditions together account for 20.7% ± 0.9% of all deaths. HIV-attributable deaths from cryptococcus, gastroenteritis, meningitis, sepsis and genitourinary conditions attributable to HIV each account for between 2% and 3% of all deaths and together they account for 11.7% ± 0.6% of all deaths. HIV-attributable deaths from Kaposi’s sarcoma, pneumocystis, anaemia, lymphoma, diseases of the skin and bone and of the digestive system each account for less than 1.5% of all deaths and together account for 3.3% ± 0.4% of all deaths.

Of the deaths included in the analysis, excluding those that were not coded or were dropped, the prevalence of HIV was 61% and the prevalence of AIDS related conditions was 69%. The HIV-attributable fraction in the whole sample was 36% and in those that were HIV-positive the HIV-attributable fraction was 60%. In 2006 the estimated number of deaths in South Africa was 691k in a study by Bradshaw *et al*. [2] and 738k in a study by the Joint United Nations Programme on HIV and AIDS (UNAIDS) [8]. If we take the average, 715k deaths, this study would suggest that the HIV-attributable deaths were about 257k, close to the 2006 estimates of 250k in the Thembisa model [2] and 270k in the Global Burden of Disease study [4,5].

## Discussion

We estimate the excess mortality from conditions known, or suspected from the literature, to be HIV/AIDS related. Our imputation of HIV status goes some way to avoiding classifying deaths by their exposure status bearing in mind that in some cases HIV/AIDS is given as the cause of death. The data in Table 1 and Fig 3 show that almost all deaths from Kaposi sarcoma, cryptococcosis or *Pneumocystis jirovecii* infections could be attributed to HIV. Kaposi sarcoma was rare before the HIV epidemic struck [14] and odds ratios for being HIV-positive compared to controls was 56 (23–176) with a DAF of 94% (92%–95%) which concurs with previous work in Soweto, Johannesburg in which the corresponding OR for being HIV-positive was 47 (32–70) [15] and 89% of patients with Kaposi sarcoma were HIV positive. Likewise cryptococcosis or *Pneumocystis jirovecii* infections were extremely rare pre-HIV, almost always occurring in persons who were immune compromised [16]. ORs for being HIV-positive for lymphomas in this analysis of 2.9 (1.7–5.0) resemble those found in a previous case control study where the OR for being HIV-positive was 5.9 (4.3–8.1) for non-Hodgkin and 1.6 (1.0–2.7) for Hodgkin lymphoma [15].

The prevalence of HIV in the control group is high and comparing this to the prevalence of HIV in ante-natal clinic surveys between 2006 and 2009 in the Johannesburg Municipality suggests an OR for being HIV-positive in our control group as compared to those in the ante-natal clinic surveys of 3.2. However, the prevalence of HIV among pregnant women in the Johannesburg Municipality may have been lower than in Soweto and it could also be that HIV causes such a force of mortality that even for deaths that are unrelated to HIV, such as car accidents or lung cancer, being HIV positive would cause additional medical complications leading to a greater case fatality. The high prevalence of HIV in older people which reaches 7% in those over 70 years of age (S1 Text, Appendix 7), is particularly striking since their life expectancy without ART is only about 2 years [17]. The problem of HIV infection in older people has been noted but only touched on briefly in the literature [14]. In the control arm of a study of newly diagnosed cancers at CHBH and Johannesburg Hospitals between 1995 and June 2004 the prevalence of HIV in those aged 65 years or more was 2.6% [18]. Various explanations have been put forward [12] but the reason for this high prevalence in older people remains unclear. As shown in S1 Text, Appendix 7, increasing the odds-ratios by a factor of 3.2 increases both the DAFs and the PAFs but the overall pattern and the general conclusions remain unchanged.

Even though the deaths examined did not cover all wards and all hospital deaths, such as deaths from cancer of the cervix, an AIDS-defining condition, and a mixture of deaths attending the intensive care unit, it is clear that the proportion of deaths in this series that are attributed to HIV are high, ranging from 20% of deaths from digestive causes to almost 100% for Kaposi sarcoma, *Pneumocystis jirovecii* and cryptococcal infection.

Tuberculosis, for which the PAF due to HIV is in excess of 90% is the most common opportunistic infection among people infected with HIV [18–20] and TB co-morbidity is very likely in Soweto where tuberculosis is endemic. Specific diagnoses of the organisms underlying meningitis, gastroenteritis and respiratory conditions were not always available and a correct diagnosis requires a culture which is costly and time consuming [21]. In sub-Saharan Africa, there is limited literature on the causative agents of meningitis, gastroenteritis and other infection related deaths but the aetiology of these is different in those with and without HIV [21].

Anti-retroviral therapy causes a decline in the incidence of several AIDS defining conditions, most clearly in the case of TB [22], but full immune recovery is only achieved if treatment is started immediately after infection when CD4+ cell counts are still high [23]. Of all the deaths between 2006 and 2009 in the medical ward of CHBH up to 36% could have been averted if people living with HIV had been started on anti-retroviral therapy early in the course of their infection. In countries with well-established treatment programs, the life expectancy for people with HIV approaches that seen in HIV-negative people [24] but in 2006–2009 the policy was not to start people on ART until their CD4+ cell-count had fallen to less than 200/*μ*L [15,18] by which time their immune system was severely compromised. In this study 6.3% of those that were HIV-positive were on ART which matches the 6% coverage of ART among South African adults at that time [10]. The mean age at death for those infected with HIV was 38 years for men and 33 years for women, suggesting that millions of life-years were lost due to the low coverage and late provision of treatment. Providing ART much earlier in the epidemic would have saved the lives of very many young adults while minimizing the economic impact of HIV, and reducing the burden on the health system [25]. A recent study of productivity losses due to premature mortality from cancer showed that the cost per cancer death in South Africa was US$101k. The 82 deaths from Kaposi’s sarcoma alone will have cost US$8.3M in lost productivity [26]. If we estimate the cost of keeping one person in CHBH for one day at about US$100 and noting that the average survival after admission was 6.7 days, then the hospital costs of the 2.5 thousand HIV-attributable deaths each year will have amounted to US$1.7 million *per annum*.

Now that ART has been provided quite widely in South Africa it will be of great importance to repeat this study in the same or a similar hospital as this will provide a direct estimate of the impact of the roll-out of ART on mortality from AIDS related conditions in South Africa.

An important strength of this study is that a confidential data sheet was used with an option for the clinician to include comments, in addition to listing the cause of death, to allow clinicians to record causes of death and express clinical opinions that may otherwise have been omitted from routine death certificates. Only consultants who were involved with the medical care of patients at CHBAH reviewed and assigned the causes of death. Laboratory results that may not have been available at the time of death such as HIV status and microbiological cultures were all reviewed at a later date and included in the data. The consultants recorded the causes of death in a manner that allowed for ICD coding without being effected by coder interpretation.

A major limitation of this study is the reliance on clinical records to establish the cause of death which is known to be prone to inaccuracies and which may be further compounded when death certificate data are used. While certain cases were discussed amongst the hospital consultants prior to arriving at a cause of death this ‘peer review and consensus’ was ad hoc and mainly confined to ‘problem’ cases.

## Supporting information

S1 Text (DOC)
