# Supplementary material for: HIV-attributable causes of death in the medical ward at the Chris Hani Baragwanath Hospital, South Africa
Source: PLoS One. 2019 May 6;14(5):e0215591. doi: 10.1371/journal.pone.0215591 (PMC6502348; doi:10.1371/journal.pone.0215591)
Supplement: S1 Text — (DOC) [file pone.0215591.s001.doc]

# Supporting Information

# Appendix 1: Data cleaning

## Dataset

Data were captured using a datasheet developed by Andrew Black in 2006 and referred to as the Baragwanath Mortality Record (BMR). Medical consultants completed the datasheet at the time of signing the deceased’s official death certificate. In some cases, the underlying cause of death reported on the BMR datasheet differed from that recorded on the official death notification form. In such cases, the cause of death was ascertained by reviewing the patient file. Data were captured in a Microsoft Excel Workbook with the cause of death coded using the Tenth International Classification of Disease codes (ICD-10). The BMR included: age, sex, date of admission, date of death, HIV status, CD4 count, ART status and underlying cause of death (Table 1).

Table 1: The main variables captured in the BMR

| Variable | Description |
| --- | --- |
| Hospital Number (ID) | Unique identifier |
| Age | Age at the time of death in years |
| Sex | Male or Female |
| Cause of Death (COD) | Clinically diagnosed disease reported to be the cause of death |
| ICD | Tenth International Classification of Diseases codes (ICD-10) |
| Date of Admission | Date at which the deceased was admitted |
| Date of Death | Date of death |
| HIV Status | HIV status of the deceased: positive, suspected, negative, unknown |
| ART Status | Status of treatment if deceased was HIV positive |

## Duplicate Patient ID numbers

In the case of duplicate patient IDs, information in other fields was inspected. If the other fields suggested that they were different individuals, a new ID was assigned, if they appeared to be the same individual only one record was kept.

## HIV and ART Status

A person’s HIV status was recorded as positive, negative, unknown, or clinically suspected because of an AIDS-defining illness. If the HIV status was not given but patients were on ART they were assumed to be HIV positive and if there was no information on ART status the HIV-status was given as ‘unknown’.

# Appendix 2: ICD codes in the data set

The ICD−10 codes in the data set were as follows:

A02−A07 Salmonella, shigellosis, amoebiasis, giardiasis

A09 Infectious gastroenteritis and colitis

A15−A16 Tuberculosis: extra-pulmonary

A17−A31 Tuberculosis: pulmonary

A32−A87 Infectious and parasitic diseases including those of the central nervous system

B01−B18 Infectious and parasitic diseases including those of the skin and viral hepatitis

B21−B24 HIV

B37−B46 Infectious and parasitic diseases: mycoses

B50−B59 Infectious and parasitic diseases: protozoal including mycoses

B69−B96 Infectious and parasitic diseases: helminthiasis and streptococcus

C02−C95 Malignant neoplasms

D00−D46 In-situ neoplasms

D51−D89 Diseases of the blood and blood-forming organs and certain disorders involving the immune mechanism

E03−E07 Disorders of the thyroid gland

E10−E88 Certain disorders involving the immune mechanism including diabetes, endocrine glands and metabolic disorder

F01−F30 Mental and behavioural disorders

G00−G08 Inflammatory diseases of the nervous system including meningitis

G10−G99 Diseases of the nervous system

H52−H70 Diseases of the eyes and ears

I05−I95 Heart and cerebro-vascular diseases

J01−J99 Respiratory system

K25−K95 Digestive system

L03−L95 Skin and subcutaneous

M01−M99 Musculoskeletal

N00−N40 Genitourinary

O90−P20 Pregnancy

Q24−Z91 Congenital, injuries poison, external causes

## Grouping the ICD codes

In order to establish control conditions, we excluded:

- Conditions for which having HIV is a risk factor for acquiring a disease which may be fatal.
- Conditions which share the same mode of transmission as HIV which may be fatal independent of HIV status but will have a higher prevalence in HIV infected persons and possibly a higher mortality.
- Diseases that may or may not be HIV related where HIV may cause the disease but there are multiple other causes that can not always be excluded.
- Diseases unrelated to HIV status but with a higher mortality in patients with HIV infection.

For the purpose of this analysis Code 0 refers to deaths that were omitted from the control group as there was uncertainty as to whether or not these causes of death were likely to be associated with HIV; code 1 refers to control conditions and codes 2 to 16 to conditions that are thought to be associated with HIV. Deaths that were not assigned an ICD code (529 or 3.4% of the sample) were excluded from the analysis. The codes were grouped as shown in Table 2.

Table 2. ICD-10 codes that were omitted, included in the control group, and included in disease categories. The right-most column is a code number corresponding to the ‘Assigned’ column in Table 3.

| Condition | ICD-10 codes from the data set | *N* |
| --- | --- | --- |
| Omitted | A02−A07; A32−A40; A49−A87; B01−B18; B3−B43; B46−B58; C22; D00−D46; H52−H70; K25−K71; K75−K95; O90, P90; T45−T60; T96; U00; U50 | 0 |
| Controls | C02−C18; C25−C45; C50−C80; C83; C90−C95; D51−D60; D68−D89; E03−E88; F01−F30; G10−G99; I05−I95; N00−N15; N18; N25−N40; Q24−Q61; S02−S72; T06−T38; T68−T87; X64−Z91 | 1 |
| Gastroenteritis | A09 | 2 |
| TB: extra pulmonary | A15−A16 | 3 |
| TB: pulmonary | A17−A19 | 4 |
| Sepsis; infectious and parasitic | A41 | 5 |
| HIV | B21−B24 | 6 |
| Pulmonary cryptococcus | B45 | 7 |
| Pneumocystis | B59−B96 | 8 |
| Kaposi’s sarcoma | C46 | 9 |
| Hodgkin’s and non-Hodgkin’s lymphoma | C81; C85 | 10 |
| Blood and blood forming organs | D61−D65 | 11 |
| Meningitis | G00−G08 | 12 |
| Pneumonia; COPD | J01−J99 | 13 |
| Digestive system | K72−K74 | 14 |
| Skin and bone | L03−L95; M01−M99 | 15 |
| Genito-urinary | N17; N19 | 16 |

Table 3 gives the data for each ICD-10 code and the last column gives the group to which each code was assigned as shown in Table 2. The grouped data are given in Table 4.

Table 3. Number of deaths, HIV-status, mean age, number of men and women, and the groups to which they are assigned as indicated in Table 2.

| ICD | Number | Negative | Positive | Unknown | Suspected | Mean age | Male | Female | Assigned |
| --- | --- | --- | --- | --- | --- | --- | --- | --- | --- |
| A02 | 24 | 1 | 21 | 1 | 1 | 39 | 14 | 0 | 0 |
| A03 | 1 | 0 | 1 | 0 | 0 | 32 | 0 | 1 | 0 |
| A06 | 8 | 1 | 5 | 2 | 0 | 52 | 3 | 5 | 0 |
| A07 | 1 | 0 | 1 | 0 | 0 | 39 | 0 | 1 | 0 |
| A09 | 509 | 19 | 366 | 61 | 63 | 43 | 220 | 289 | 2 |
| A15 | 685 | 18 | 565 | 48 | 54 | 39 | 367 | 318 | 3 |
| A16 | 736 | 38 | 557 | 64 | 77 | 41 | 394 | 342 | 3 |
| A17 | 364 | 10 | 295 | 25 | 34 | 38 | 197 | 318 | 4 |
| A18 | 473 | 17 | 394 | 23 | 39 | 39 | 246 | 342 | 4 |
| A19 | 95 | 4 | 82 | 3 | 6 | 39 | 49 | 46 | 4 |
| A31 | 17 | 0 | 16 | 0 | 1 | 39 | 10 | 7 | 4 |
| A32 | 1 | 1 | 0 | 0 | 0 | 28 | 1 | 0 | 0 |
| A35 | 2 | 0 | 0 | 2 | 0 | 42 | 2 | 0 | 0 |
| A39 | 3 | 0 | 2 | 1 | 0 | 31 | 1 | 2 | 0 |
| A40 | 4 | 0 | 1 | 2 | 1 | 54 | 3 | 1 | 0 |
| A41 | 889 | 152 | 454 | 194 | 89 | 49 | 386 | 503 | 5 |
| A49 | 2 | 0 | 2 | 0 | 0 | 41 | 1 | 1 | 0 |
| A52 | 1 | 0 | 0 | 1 | 0 | 32 | 1 | 0 | 0 |
| A81 | 6 | 0 | 6 | 0 | 0 | 36 | 3 | 3 | 0 |
| A85 | 1 | 0 | 1 | 0 | 0 | 32 | 0 | 1 | 0 |
| A86 | 1 | 0 | 0 | 0 | 1 | 36 | 1 | 0 | 0 |
| A87 | 1 | 0 | 1 | 0 | 0 | 36 | 0 | 1 | 0 |
| B01 | 20 | 1 | 11 | 4 | 4 | 30 | 4 | 16 | 0 |
| B02 | 1 | 0 | 1 | 0 | 0 | 34 | 0 | 1 | 0 |
| B10 | 7 | 0 | 4 | 2 | 1 | 47 | 2 | 5 | 0 |
| B16 | 9 | 2 | 5 | 2 | 0 | 42 | 4 | 5 | 0 |
| B17 | 2 | 0 | 1 | 1 | 0 | 47 | 1 | 1 | 0 |
| B18 | 1 | 1 | 0 | 0 | 0 | 57 | 1 | 0 | 0 |
| B21 | 4 | 0 | 4 | 0 | 0 | 31 | 2 | 2 | 6 |
| B22 | 6 | 2 | 3 | 0 | 1 | 42 | 3 | 3 | 6 |
| B23 | 7 | 0 | 7 | 0 | 0 | 35 | 4 | 3 | 6 |
| B24 | 656 | 5 | 601 | 5 | 45 | 39 | 319 | 337 | 6 |
| B37 | 2 | 0 | 1 | 1 | 0 | 70 | 0 | 2 | 0 |
| B39 | 2 | 0 | 2 | 0 | 0 | 31 | 2 | 0 | 0 |
| B42 | 1 | 0 | 1 | 0 | 0 | 28 | 0 | 1 | 0 |
| B43 | 17 | 5 | 9 | 2 | 1 | 40 | 11 | 6 | 0 |
| B45 | 353 | 1 | 323 | 6 | 23 | 37 | 168 | 185 | 7 |
| B46 | 1 | 0 | 0 | 1 | 0 | 57 | 0 | 1 | 0 |
| B50 | 3 | 0 | 0 | 2 | 1 | 39 | 2 | 1 | 0 |
| B54 | 9 | 2 | 2 | 3 | 2 | 41 | 4 | 5 | 0 |
| B58 | 5 | 0 | 5 | 0 | 0 | 39 | 4 | 1 | 0 |
| B59 | 170 | 2 | 149 | 6 | 13 | 37 | 60 | 110 | 8 |
| B69 | 1 | 0 | 1 | 0 | 0 | 61 | 0 | 1 | 8 |
| B95 | 6 | 0 | 6 | 0 | 0 | 48 | 3 | 3 | 8 |
| B96 | 43 | 6 | 28 | 6 | 3 | 48 | 21 | 22 | 8 |
| C02 | 1 | 0 | 0 | 1 | 0 | 59 | 1 | 0 | 1 |
| C04 | 1 | 0 | 1 | 0 | 0 | 48 | 0 | 1 | 1 |
| C10 | 1 | 0 | 0 | 1 | 0 | 45 | 1 | 0 | 1 |
| C15 | 4 | 1 | 0 | 2 | 1 | 73 | 4 | 0 | 1 |
| C16 | 1 | 0 | 0 | 1 | 0 | 67 | 1 | 0 | 1 |
| C18 | 1 | 0 | 1 | 0 | 0 | 55 | 1 | 0 | 1 |
| C22 | 34 | 9 | 5 | 14 | 6 | 60 | 20 | 14 | 0 |
| C25 | 15 | 3 | 0 | 10 | 2 | 62 | 7 | 8 | 1 |
| C26 | 1 | 0 | 0 | 1 | 0 | 69 | 1 | 0 | 1 |
| C32 | 2 | 0 | 0 | 2 | 0 | 66 | 2 | 0 | 1 |
| C34 | 151 | 86 | 9 | 45 | 11 | 61 | 121 | 30 | 1 |
| C45 | 2 | 1 | 0 | 1 | 0 | 65 | 1 | 1 | 1 |
| C46 | 117 | 3 | 108 | 2 | 4 | 37 | 63 | 54 | 9 |
| C50 | 34 | 9 | 7 | 13 | 5 | 59 | 0 | 34 | 1 |
| C56 | 6 | 1 | 1 | 3 | 1 | 58 | 0 | 6 | 1 |
| C61 | 58 | 26 | 0 | 24 | 8 | 72 | 58 | 0 | 1 |
| C62 | 2 | 1 | 0 | 1 | 0 | 35 | 2 | 0 | 1 |
| C64 | 4 | 2 | 1 | 1 | 0 | 52 | 3 | 1 | 1 |
| C67 | 7 | 2 | 0 | 3 | 2 | 63 | 7 | 0 | 1 |
| C70 | 4 | 0 | 0 | 4 | 0 | 61 | 1 | 3 | 1 |
| C71 | 3 | 1 | 0 | 2 | 0 | 52 | 1 | 2 | 1 |
| C72 | 1 | 0 | 1 | 0 | 0 | 31 | 0 | 1 | 1 |
| C74 | 1 | 0 | 1 | 0 | 0 | 32 | 1 | 0 | 1 |
| C76 | 2 | 0 | 0 | 2 | 0 | 68 | 2 | 0 | 1 |
| C78 | 1 | 1 | 0 | 0 | 0 | 73 | 1 | 0 | 1 |
| C79 | 25 | 10 | 0 | 10 | 5 | 64 | 17 | 8 | 1 |
| C80 | 95 | 39 | 7 | 40 | 9 | 65 | 42 | 53 | 1 |
| C81 | 41 | 12 | 25 | 4 | 0 | 40 | 19 | 22 | 10 |
| C83 | 2 | 1 | 0 | 1 | 0 | 57 | 2 | 0 | 1 |
| C85 | 54 | 5 | 44 | 3 | 2 | 40 | 28 | 26 | 10 |
| C90 | 40 | 20 | 3 | 12 | 5 | 61 | 18 | 22 | 1 |
| C91 | 17 | 8 | 3 | 6 | 0 | 59 | 8 | 9 | 1 |
| C92 | 29 | 12 | 3 | 11 | 3 | 49 | 12 | 17 | 1 |
| C94 | 3 | 1 | 1 | 1 | 0 | 67 | 2 | 1 | 1 |
| C95 | 12 | 6 | 2 | 3 | 1 | 40 | 9 | 3 | 1 |
| D00 | 19 | 8 | 1 | 4 | 6 | 62 | 13 | 6 | 0 |
| D01 | 9 | 1 | 4 | 4 | 0 | 59 | 5 | 4 | 0 |
| D02 | 1 | 0 | 0 | 0 | 1 | 94 | 1 | 0 | 0 |
| D03 | 2 | 1 | 0 | 1 | 0 | 67 | 1 | 1 | 0 |
| D06 | 29 | 2 | 11 | 12 | 4 | 57 | 0 | 29 | 0 |
| D07 | 6 | 1 | 1 | 2 | 2 | 68 | 0 | 6 | 0 |
| D09 | 1 | 0 | 0 | 1 | 0 | 83 | 1 | 0 | 0 |
| D15 | 1 | 1 | 0 | 0 | 0 | 20 | 0 | 1 | 0 |
| D43 | 2 | 0 | 1 | 1 | 0 | 49 | 0 | 2 | 0 |
| D46 | 1 | 1 | 0 | 0 | 0 | 85 | 0 | 1 | 0 |
| D51 | 2 | 2 | 0 | 0 | 0 | 51 | 0 | 2 | 1 |
| D53 | 1 | 0 | 0 | 1 | 0 | 63 | 0 | 1 | 1 |
| D59 | 1 | 1 | 0 | 0 | 0 | 28 | 0 | 1 | 1 |
| D60 | 2 | 0 | 2 | 0 | 0 | 39 | 0 | 2 | 1 |
| D61 | 37 | 4 | 31 | 0 | 2 | 36 | 24 | 13 | 11 |
| D64 | 85 | 6 | 51 | 20 | 8 | 43 | 32 | 53 | 11 |
| D65 | 14 | 2 | 10 | 2 | 0 | 37 | 5 | 9 | 11 |
| D68 | 20 | 13 | 1 | 5 | 1 | 38 | 4 | 16 | 1 |
| D69 | 15 | 1 | 12 | 1 | 1 | 41 | 5 | 10 | 1 |
| D70 | 3 | 0 | 1 | 2 | 0 | 54 | 1 | 2 | 1 |
| D73 | 1 | 0 | 1 | 0 | 0 | 40 | 0 | 1 | 1 |
| D75 | 1 | 0 | 1 | 0 | 0 | 51 | 0 | 1 | 1 |
| D81 | 1 | 0 | 0 | 0 | 1 | 65 | 1 | 0 | 1 |
| D89 | 6 | 0 | 5 | 0 | 1 | 35 | 0 | 6 | 1 |
| E03 | 5 | 2 | 0 | 3 | 0 | 78 | 2 | 3 | 1 |
| E05 | 3 | 0 | 0 | 3 | 0 | 52 | 1 | 2 | 1 |
| E07 | 1 | 1 | 0 | 0 | 0 | 49 | 1 | 0 | 1 |
| E10 | 3 | 0 | 0 | 2 | 1 | 75 | 0 | 3 | 1 |
| E11 | 11 | 3 | 1 | 7 | 0 | 63 | 3 | 8 | 1 |
| E12 | 24 | 8 | 1 | 10 | 5 | 68 | 11 | 13 | 1 |
| E14 | 164 | 52 | 14 | 73 | 25 | 60 | 64 | 100 | 1 |
| E15 | 5 | 1 | 0 | 4 | 0 | 70 | 2 | 3 | 1 |
| E16 | 134 | 25 | 29 | 59 | 21 | 58 | 67 | 67 | 1 |
| E20 | 3 | 0 | 0 | 3 | 0 | 64 | 3 | 0 | 1 |
| E21 | 7 | 3 | 1 | 3 | 0 | 66 | 5 | 2 | 1 |
| E22 | 2 | 0 | 1 | 0 | 1 | 39 | 2 | 0 | 1 |
| E23 | 1 | 0 | 0 | 1 | 0 | 54 | 1 | 0 | 1 |
| E27 | 4 | 0 | 4 | 0 | 0 | 38 | 2 | 2 | 1 |
| E46 | 2 | 0 | 0 | 2 | 0 | 57 | 1 | 1 | 1 |
| E52 | 1 | 0 | 0 | 1 | 0 | 38 | 0 | 1 | 1 |
| E66 | 10 | 4 | 1 | 4 | 1 | 59 | 2 | 8 | 1 |
| E77 | 3 | 1 | 0 | 1 | 1 | 62 | 1 | 2 | 1 |
| E84 | 1 | 0 | 1 | 0 | 0 | 33 | 1 | 0 | 1 |
| E86 | 81 | 8 | 37 | 18 | 18 | 51 | 48 | 33 | 1 |
| E87 | 142 | 5 | 117 | 12 | 8 | 41 | 34 | 108 | 1 |
| E88 | 6 | 2 | 2 | 1 | 1 | 63 | 3 | 3 | 1 |
| F01 | 10 | 4 | 1 | 4 | 1 | 73 | 6 | 4 | 1 |
| F02 | 8 | 0 | 7 | 1 | 0 | 47 | 3 | 5 | 1 |
| F03 | 15 | 4 | 5 | 6 | 0 | 63 | 7 | 8 | 1 |
| F05 | 12 | 0 | 4 | 5 | 3 | 57 | 7 | 5 | 1 |
| F07 | 1 | 1 | 0 | 0 | 0 | 66 | 0 | 1 | 1 |
| F10 | 1 | 0 | 1 | 0 | 0 | 49 | 1 | 0 | 1 |
| F30 | 1 | 0 | 1 | 0 | 0 | 29 | 0 | 1 | 1 |
| G00 | 79 | 5 | 46 | 17 | 11 | 40 | 31 | 48 | 12 |
| G03 | 593 | 24 | 388 | 109 | 72 | 40 | 294 | 299 | 12 |
| G04 | 5 | 0 | 5 | 0 | 0 | 46 | 3 | 2 | 12 |
| G06 | 5 | 1 | 2 | 1 | 1 | 48 | 2 | 3 | 12 |
| G08 | 2 | 0 | 1 | 1 | 0 | 54 | 0 | 2 | 12 |
| G10 | 1 | 1 | 0 | 0 | 0 | 51 | 0 | 1 | 1 |
| G11 | 2 | 0 | 0 | 2 | 0 | 50 | 2 | 0 | 1 |
| G12 | 2 | 1 | 0 | 1 | 0 | 52 | 2 | 0 | 1 |
| G20 | 3 | 2 | 0 | 1 | 0 | 76 | 3 | 0 | 1 |
| G21 | 3 | 0 | 1 | 2 | 0 | 29 | 2 | 1 | 1 |
| G23 | 1 | 0 | 0 | 1 | 0 | 90 | 0 | 1 | 1 |
| G30 | 2 | 1 | 0 | 1 | 0 | 75 | 1 | 1 | 1 |
| G31 | 1 | 0 | 0 | 1 | 0 | 54 | 1 | 0 | 1 |
| G37 | 10 | 0 | 6 | 4 | 0 | 44 | 5 | 5 | 1 |
| G40 | 55 | 10 | 13 | 21 | 11 | 49 | 38 | 17 | 1 |
| G41 | 32 | 7 | 4 | 18 | 3 | 50 | 17 | 15 | 1 |
| G45 | 2 | 1 | 0 | 1 | 0 | 68 | 1 | 1 | 1 |
| G47 | 2 | 1 | 0 | 1 | 0 | 72 | 1 | 1 | 1 |
| G58 | 1 | 0 | 0 | 0 | 1 | 54 | 1 | 0 | 1 |
| G61 | 7 | 0 | 4 | 3 | 0 | 43 | 3 | 4 | 1 |
| G70 | 1 | 1 | 0 | 0 | 0 | 35 | 0 | 1 | 1 |
| G71 | 1 | 0 | 0 | 1 | 0 | 15 | 1 | 0 | 1 |
| G72 | 1 | 0 | 1 | 0 | 0 | 27 | 1 | 0 | 1 |
| G81 | 12 | 3 | 3 | 3 | 3 | 60 | 5 | 7 | 1 |
| G82 | 6 | 1 | 1 | 3 | 1 | 60 | 2 | 4 | 1 |
| G83 | 1 | 1 | 0 | 0 | 0 | 82 | 0 | 1 | 1 |
| G90 | 2 | 0 | 2 | 0 | 0 | 62 | 0 | 2 | 1 |
| G91 | 16 | 2 | 9 | 2 | 3 | 42 | 8 | 8 | 1 |
| G93 | 26 | 4 | 9 | 12 | 1 | 46 | 13 | 13 | 1 |
| G94 | 1 | 1 | 0 | 0 | 0 | 51 | 1 | 0 | 1 |
| G95 | 3 | 0 | 3 | 0 | 0 | 40 | 1 | 2 | 1 |
| G99 | 2 | 1 | 1 | 0 | 0 | 34 | 0 | 2 | 1 |
| H52 | 1 | 0 | 1 | 0 | 0 | 34 | 1 | 0 | 0 |
| H66 | 1 | 0 | 1 | 0 | 0 | 44 | 0 | 1 | 0 |
| H70 | 1 | 0 | 1 | 0 | 0 | 47 | 0 | 1 | 0 |
| I05 | 13 | 6 | 1 | 6 | 0 | 55 | 2 | 11 | 1 |
| I06 | 2 | 2 | 0 | 0 | 0 | 51 | 1 | 1 | 1 |
| i08 | 6 | 3 | 0 | 3 | 0 | 40 | 3 | 3 | 1 |
| I09 | 8 | 5 | 1 | 1 | 1 | 51 | 4 | 4 | 1 |
| I10 | 106 | 25 | 6 | 60 | 15 | 65 | 33 | 73 | 1 |
| I11 | 327 | 123 | 25 | 138 | 41 | 65 | 113 | 214 | 1 |
| I21 | 40 | 11 | 3 | 20 | 6 | 63 | 22 | 18 | 1 |
| I22 | 2 | 1 | 0 | 1 | 0 | 70 | 2 | 0 | 1 |
| I24 | 5 | 2 | 0 | 3 | 0 | 71 | 1 | 4 | 1 |
| I25 | 12 | 4 | 0 | 6 | 2 | 67 | 6 | 6 | 1 |
| I26 | 115 | 30 | 37 | 39 | 9 | 51 | 35 | 80 | 1 |
| I27 | 198 | 62 | 37 | 84 | 15 | 59 | 96 | 102 | 1 |
| I30 | 3 | 1 | 2 | 0 | 0 | 35 | 2 | 1 | 1 |
| I31 | 15 | 3 | 5 | 3 | 4 | 40 | 8 | 7 | 1 |
| I33 | 2 | 1 | 1 | 0 | 0 | 25 | 1 | 1 | 1 |
| I35 | 1 | 0 | 0 | 0 | 1 | 51 | 0 | 1 | 1 |
| I37 | 2 | 2 | 0 | 0 | 0 | 41 | 0 | 2 | 1 |
| I38 | 12 | 3 | 4 | 3 | 2 | 45 | 6 | 6 | 1 |
| I42 | 231 | 58 | 60 | 106 | 7 | 53 | 116 | 115 | 1 |
| I44 | 7 | 1 | 0 | 5 | 1 | 65 | 2 | 5 | 1 |
| I46 | 3 | 2 | 0 | 1 | 0 | 93 | 0 | 3 | 1 |
| I47 | 5 | 2 | 0 | 3 | 0 | 65 | 3 | 2 | 1 |
| I48 | 14 | 4 | 1 | 6 | 3 | 62 | 4 | 10 | 1 |
| I49 | 1 | 1 | 0 | 0 | 0 | 68 | 0 | 1 | 1 |
| I50 | 318 | 105 | 36 | 143 | 34 | 62 | 123 | 195 | 1 |
| I51 | 5 | 0 | 1 | 4 | 0 | 49 | 3 | 2 | 1 |
| I60 | 1 | 0 | 0 | 1 | 0 | 51 | 0 | 1 | 1 |
| I61 | 202 | 48 | 20 | 119 | 15 | 57 | 97 | 105 | 1 |
| I62 | 25 | 6 | 3 | 16 | 0 | 57 | 16 | 9 | 1 |
| I63 | 38 | 7 | 11 | 15 | 5 | 62 | 16 | 22 | 1 |
| I64 | 998 | 331 | 108 | 461 | 98 | 64 | 390 | 608 | 1 |
| I71 | 4 | 1 | 1 | 2 | 0 | 53 | 1 | 3 | 1 |
| I72 | 1 | 0 | 0 | 1 | 0 | 87 | 1 | 0 | 1 |
| I73 | 7 | 2 | 1 | 4 | 0 | 63 | 2 | 5 | 1 |
| I80 | 22 | 2 | 13 | 6 | 1 | 48 | 10 | 12 | 1 |
| I82 | 3 | 1 | 2 | 0 | 0 | 30 | 1 | 2 | 1 |
| I85 | 2 | 1 | 0 | 1 | 0 | 66 | 0 | 2 | 1 |
| I88 | 1 | 0 | 0 | 0 | 1 | 25 | 0 | 1 | 1 |
| I95 | 3 | 1 | 1 | 1 | 0 | 50 | 2 | 1 | 1 |
| J01 | 1 | 0 | 1 | 0 | 0 | 31 | 0 | 1 | 13 |
| J06 | 2 | 0 | 2 | 0 | 0 | 29 | 0 | 2 | 13 |
| J13 | 4 | 1 | 3 | 0 | 0 | 45 | 2 | 2 | 13 |
| J15 | 4 | 0 | 2 | 1 | 1 | 53 | 3 | 1 | 13 |
| J17 | 1 | 0 | 1 | 0 | 0 | 29 | 0 | 1 | 13 |
| J18 | 2198 | 210 | 1206 | 453 | 329 | 46 | 1047 | 1151 | 13 |
| J22 | 233 | 11 | 140 | 38 | 44 | 45 | 126 | 107 | 13 |
| J35 | 2 | 1 | 1 | 0 | 0 | 44 | 0 | 2 | 13 |
| J43 | 2 | 1 | 1 | 0 | 0 | 58 | 1 | 1 | 13 |
| J44 | 126 | 29 | 5 | 76 | 16 | 63 | 98 | 28 | 13 |
| J45 | 2 | 0 | 1 | 0 | 1 | 52 | 1 | 1 | 13 |
| J46 | 23 | 6 | 4 | 9 | 4 | 57 | 11 | 12 | 13 |
| J47 | 22 | 10 | 8 | 4 | 0 | 47 | 13 | 9 | 13 |
| J69 | 40 | 6 | 5 | 25 | 4 | 52 | 27 | 13 | 13 |
| J81 | 19 | 5 | 1 | 9 | 4 | 56 | 9 | 10 | 13 |
| J84 | 5 | 3 | 1 | 0 | 1 | 63 | 2 | 3 | 13 |
| J85 | 15 | 4 | 7 | 3 | 1 | 45 | 11 | 4 | 13 |
| J86 | 37 | 5 | 29 | 3 | 0 | 39 | 20 | 17 | 13 |
| J90 | 62 | 16 | 24 | 12 | 10 | 49 | 33 | 29 | 13 |
| J91 | 2 | 0 | 1 | 1 | 0 | 36 | 1 | 1 | 13 |
| J93 | 20 | 2 | 13 | 3 | 2 | 47 | 16 | 4 | 13 |
| J94 | 2 | 0 | 2 | 0 | 0 | 39 | 0 | 2 | 13 |
| J96 | 31 | 10 | 9 | 6 | 6 | 53 | 20 | 11 | 13 |
| J98 | 3 | 1 | 1 | 0 | 1 | 62 | 2 | 1 | 13 |
| J99 | 2 | 0 | 1 | 1 | 0 | 50 | 1 | 1 | 13 |
| K25 | 4 | 1 | 0 | 3 | 0 | 48 | 4 | 0 | 0 |
| K27 | 6 | 3 | 0 | 3 | 0 | 68 | 2 | 4 | 0 |
| K29 | 3 | 0 | 2 | 1 | 0 | 55 | 2 | 1 | 0 |
| K31 | 2 | 0 | 1 | 1 | 0 | 28 | 1 | 1 | 0 |
| K42 | 1 | 1 | 0 | 0 | 0 | 80 | 0 | 1 | 0 |
| K56 | 9 | 1 | 1 | 5 | 2 | 55 | 3 | 6 | 0 |
| K59 | 13 | 2 | 10 | 1 | 0 | 45 | 5 | 8 | 0 |
| K63 | 1 | 0 | 1 | 0 | 0 | 20 | 0 | 1 | 0 |
| K65 | 13 | 4 | 3 | 6 | 0 | 47 | 7 | 6 | 0 |
| K70 | 24 | 6 | 0 | 15 | 3 | 55 | 13 | 11 | 0 |
| K71 | 28 | 1 | 24 | 2 | 1 | 35 | 16 | 12 | 0 |
| K72 | 165 | 23 | 63 | 62 | 17 | 49 | 96 | 69 | 14 |
| K73 | 34 | 0 | 21 | 9 | 4 | 38 | 18 | 16 | 14 |
| K74 | 41 | 12 | 9 | 19 | 1 | 51 | 27 | 14 | 14 |
| K75 | 2 | 2 | 0 | 0 | 0 | 39 | 0 | 2 | 0 |
| K76 | 18 | 3 | 6 | 7 | 2 | 48 | 15 | 3 | 0 |
| K77 | 1 | 0 | 1 | 0 | 0 | 40 | 1 | 0 | 0 |
| K83 | 1 | 0 | 0 | 1 | 0 | 81 | 0 | 1 | 0 |
| K85 | 7 | 0 | 4 | 3 | 0 | 49 | 3 | 4 | 0 |
| K86 | 1 | 1 | 0 | 0 | 0 | 47 | 1 | 0 | 0 |
| K92 | 67 | 18 | 12 | 31 | 6 | 59 | 34 | 33 | 0 |
| K95 | 1 | 0 | 1 | 0 | 0 | 33 | 0 | 1 | 0 |
| L03 | 4 | 1 | 1 | 2 | 0 | 55 | 1 | 3 | 15 |
| L12 | 2 | 2 | 0 | 0 | 0 | 66 | 1 | 1 | 15 |
| L13 | 4 | 0 | 1 | 3 | 0 | 53 | 3 | 1 | 15 |
| L30 | 1 | 0 | 1 | 0 | 0 | 48 | 0 | 1 | 15 |
| L51 | 29 | 1 | 21 | 3 | 4 | 45 | 18 | 11 | 15 |
| L53 | 1 | 0 | 1 | 0 | 0 | 45 | 0 | 1 | 15 |
| L56 | 1 | 0 | 1 | 0 | 0 | 35 | 0 | 1 | 15 |
| L89 | 3 | 1 | 0 | 2 | 0 | 54 | 1 | 2 | 15 |
| L95 | 1 | 0 | 0 | 1 | 0 | 39 | 0 | 1 | 15 |
| M01 | 1 | 0 | 1 | 0 | 0 | 38 | 1 | 0 | 15 |
| M07 | 1 | 0 | 0 | 1 | 0 | 63 | 0 | 1 | 15 |
| M19 | 1 | 1 | 0 | 0 | 0 | 57 | 0 | 1 | 15 |
| M31 | 14 | 3 | 8 | 1 | 2 | 35 | 3 | 11 | 15 |
| M33 | 2 | 1 | 0 | 1 | 0 | 60 | 1 | 1 | 15 |
| M34 | 3 | 1 | 0 | 1 | 1 | 32 | 0 | 3 | 15 |
| M35 | 1 | 0 | 1 | 0 | 0 | 33 | 0 | 1 | 15 |
| M49 | 1 | 0 | 1 | 0 | 0 | 22 | 0 | 1 | 15 |
| M72 | 2 | 1 | 0 | 1 | 0 | 49 | 1 | 1 | 15 |
| M80 | 12 | 4 | 4 | 4 | 0 | 53 | 8 | 4 | 15 |
| M81 | 54 | 21 | 8 | 19 | 6 | 58 | 36 | 18 | 15 |
| M83 | 1 | 1 | 0 | 0 | 0 | 47 | 1 | 0 | 15 |
| M85 | 1 | 0 | 0 | 0 | 1 | 45 | 1 | 0 | 15 |
| M86 | 1 | 0 | 1 | 0 | 0 | 46 | 0 | 1 | 15 |
| M89 | 1 | 0 | 0 | 0 | 1 | 22 | 1 | 0 | 15 |
| M98 | 3 | 1 | 1 | 1 | 0 | 41 | 3 | 0 | 15 |
| M99 | 2 | 0 | 2 | 0 | 0 | 35 | 1 | 1 | 15 |
| N00 | 3 | 1 | 1 | 1 | 0 | 37 | 2 | 1 | 1 |
| N04 | 6 | 2 | 3 | 1 | 0 | 27 | 1 | 5 | 1 |
| N05 | 1 | 0 | 0 | 1 | 0 | 58 | 0 | 1 | 1 |
| N11 | 5 | 2 | 1 | 1 | 1 | 44 | 2 | 3 | 1 |
| N13 | 11 | 5 | 1 | 5 | 0 | 76 | 9 | 2 | 1 |
| N14 | 2 | 1 | 0 | 0 | 1 | 57 | 2 | 0 | 1 |
| N15 | 1 | 0 | 1 | 0 | 0 | 61 | 1 | 0 | 1 |
| N17 | 153 | 28 | 83 | 28 | 14 | 49 | 77 | 76 | 16 |
| N18 | 13 | 6 | 3 | 2 | 2 | 50 | 7 | 6 | 1 |
| N19 | 722 | 161 | 295 | 193 | 73 | 51 | 384 | 338 | 16 |
| N25 | 2 | 2 | 0 | 0 | 0 | 61 | 0 | 2 | 1 |
| N39 | 10 | 3 | 0 | 4 | 3 | 76 | 3 | 7 | 1 |
| NA0 | 45 | 6 | 17 | 19 | 3 | 49 | 25 | 20 | 1 |
| O90 | 17 | 4 | 7 | 4 | 2 | 29 | 0 | 17 | 0 |
| P20 | 1 | 0 | 0 | 0 | 1 | 29 | 0 | 1 | 0 |
| Q24 | 1 | 0 | 1 | 0 | 0 | 24 | 0 | 1 | 1 |
| Q26 | 4 | 2 | 0 | 2 | 0 | 56 | 4 | 0 | 1 |
| Q61 | 1 | 1 | 0 | 0 | 0 | 56 | 1 | 0 | 1 |
| S02 | 1 | 0 | 0 | 1 | 0 | 14 | 1 | 0 | 1 |
| S06 | 72 | 19 | 4 | 45 | 4 | 53 | 27 | 45 | 1 |
| S72 | 1 | 0 | 0 | 1 | 0 | 63 | 1 | 0 | 1 |
| T06 | 1 | 0 | 0 | 0 | 1 | 35 | 1 | 0 | 1 |
| T38 | 1 | 0 | 0 | 1 | 0 | 38 | 0 | 1 | 1 |
| T45 | 8 | 2 | 0 | 6 | 0 | 59 | 3 | 5 | 0 |
| T48 | 1 | 0 | 0 | 1 | 0 | 21 | 0 | 1 | 0 |
| T59 | 1 | 0 | 0 | 1 | 0 | 67 | 1 | 0 | 0 |
| T60 | 27 | 1 | 1 | 20 | 5 | 33 | 17 | 10 | 0 |
| T68 | 11 | 2 | 1 | 7 | 1 | 63 | 4 | 7 | 1 |
| T78 | 4 | 1 | 1 | 2 | 0 | 56 | 0 | 4 | 1 |
| T87 | 1 | 0 | 0 | 1 | 0 | 76 | 0 | 1 | 1 |
| T96 | 4 | 1 | 1 | 2 | 0 | 44 | 2 | 2 | 0 |
| U00 | 1 | 0 | 0 | 0 | 1 | 70 | 0 | 1 | 0 |
| U50 | 21 | 1 | 16 | 1 | 3 | 38 | 10 | 11 | 0 |
| X64 | 3 | 0 | 0 | 3 | 0 | 29 | 2 | 1 | 1 |
| X66 | 1 | 0 | 1 | 0 | 0 | 26 | 0 | 1 | 1 |
| X69 | 1 | 0 | 0 | 1 | 0 | 34 | 0 | 1 | 1 |
| Y84 | 1 | 0 | 0 | 0 | 1 | 54 | 1 | 0 | 1 |
| Y91 | 9 | 2 | 0 | 7 | 0 | 59 | 6 | 3 | 1 |
| Y95 | 1 | 0 | 0 | 1 | 0 | 79 | 1 | 0 | 1 |
| Z03 | 91 | 3 | 71 | 12 | 5 | 41 | 50 | 41 | 1 |
| Z49 | 1 | 0 | 0 | 1 | 0 | 48 | 1 | 0 | 1 |
| Z91 | 1 | 0 | 0 | 1 | 0 | 54 | 0 | 1 | 1 |
| UC | 652 | 104 | 184 | 167 | 74 | 49 | 310 | 219 | −1 |

Table 4. The data grouped as indicated in Table 3. The columns give the number of deaths in each category, the proportion of all deaths attributable to each category, the number in each HIV-category (negative, positive, unknown or suspected), the prevalence of HIV assuming that all that are HIV-unknown are uninfected and all that are suspected of being infected with HIV are infected, the mean age, the number of men and women and the sex ratio.

|  | |  | Prop. | HIV status | | | | Prev. | Age |  |  |  |
| --- | --- | --- | --- | --- | --- | --- | --- | --- | --- | --- | --- | --- |
| Category | | No. | (%) | Neg. | Pos. | Unkn. | Susp. | (%) | (yrs) | Male | Female | $\frac{F}{F+M}$ |
| -1 | Not coded | 529 | 3.4 | 104 | 184 | 167 | 74 | 49 | 52 | 310 | 219 | 0.41 |
| 0 | Dropped | 527 | 3.4 | 90 | 200 | 180 | 57 | 49 | 48 | 247 | 270 | 0.52 |
| 1 | Controls | 4503 | 28.6 | 1325 | 835 | 1894 | 449 | 29 | 59 | 1998 | 2505 | 0.56 |
| 2 | Gastric | 509 | 3.2 | 19 | 366 | 61 | 63 | 84 | 43 | 220 | 289 | 0.57 |
| 3 | TB: extra pulmon. | 1421 | 9.0 | 56 | 1122 | 112 | 131 | 88 | 40 | 761 | 660 | 0.46 |
| 4 | TB: pulmonary | 949 | 6.0 | 31 | 787 | 51 | 80 | 91 | 39 | 502 | 713 | 0.59 |
| 5 | Other sepsis | 889 | 5.7 | 152 | 454 | 194 | 89 | 61 | 49 | 386 | 503 | 0.57 |
| 6 | HIV | 673 | 4.3 | 7 | 615 | 5 | 46 | 98 | 39 | 328 | 345 | 0.51 |
| 7 | Pulm. cryptococcus | 353 | 2.2 | 1 | 323 | 6 | 23 | 98 | 37 | 168 | 185 | 0.52 |
| 8 | Pneumocystis | 220 | 1.4 | 8 | 184 | 12 | 16 | 91 | 40 | 84 | 136 | 0.62 |
| 9 | Kaposi’s sarcoma | 117 | 0.7 | 3 | 108 | 2 | 4 | 96 | 37 | 63 | 54 | 0.46 |
| 10 | Hodgkin’s | 95 | 0.6 | 17 | 69 | 7 | 2 | 75 | 40 | 47 | 48 | 0.51 |
| 11 | Blood | 136 | 0.9 | 12 | 92 | 22 | 10 | 75 | 41 | 61 | 75 | 0.55 |
| 12 | Meningitis | 684 | 4.3 | 30 | 442 | 128 | 84 | 77 | 40 | 330 | 354 | 0.52 |
| 13 | Respiratory | 2858 | 18.2 | 321 | 1469 | 644 | 424 | 66 | 47 | 1444 | 1414 | 0.49 |
| 14 | Digestive | 240 | 1.5 | 35 | 93 | 90 | 22 | 48 | 48 | 141 | 99 | 0.41 |
| 15 | Skin and bone | 147 | 0.9 | 39 | 53 | 40 | 15 | 46 | 50 | 81 | 66 | 0.45 |
| 16 | Genito-urinary | 875 | 5.6 | 189 | 378 | 221 | 87 | 53 | 51 | 461 | 414 | 0.47 |

# Appendix 3: Distribution of cases by age, sex and HIV-status

Each person that died was recorded as being HIV positive (*P*), HIV-negative (*N*), HIV suspected (*S*) or HIV unknown (*U*). For those that were positive or negative we fitted the distribution of cases by age and gender to skew-normal distributions so that *P*(*a*) the number of people of HIV-positive people of age *a*, for each gender is given by

$$P(a)=\frac{\bar{P}}{\sigma\pi}\mathrm{e}^{-(a-\mu)^{2}/2\sigma^{2}}\int_{-\infty}^{\alpha(a-\mu)/\sigma} \mathrm{e}^{-t^{2}/2}dt$$

where $\bar{P}$ is the total number of people recorded as being HIV-positive, *μ* is the location parameter, *σ* is the scale parameter, and *α* determines the skewness of the distribution. A similar functional form is fitted to those that are recorded as being HIV-negative, *N*(*a*). The data and the fitted curves are plotted in Figure 1.

Figure 1. A and B: men; C and D women. Brown: HIV-negative; green: HIV unknown. Blue: HIV-positive; red: HIV-suspected.

The data in Figure 1 allow us to estimate the proportion of men and women who are HIV-positive and HIV-negative among those whose status is suspected or unknown by fitting a weighted sum of the fitted curves for those whose status is known to be positive or negative to those whose status is unknown or suspected. We then have

$$S(a)=\pi\frac{P(a)}{\bar{P}}+\nu\frac{N(a)}{\bar{N}}$$

For those suspected of being HIV-positive *π * is the estimated number that are HIV-positive, *ν* is the estimated number that are HIV negative. A similar expression for *U*(*a*) gives the corresponding estimates for those whose HIV-status is unknown. We do this separately for men and women. The data and the fitted curves are given in Figure 1 and Table 5.

Table 5. Parameters for the skew-normal fits to the number of men (M) and women (W) who are HIV positive (P) or HIV negative (N).

|  | MP | MN | WP | WN |
| --- | --- | --- | --- | --- |
| Shape | 2.69 | −2.16 | 3.51 | −4.25 |
| Norm | 3672 | 1155 | 4102 | 1285 |
| Location | 29.9 | 76.4 | 24.9 | 85.1 |
| Scale | 14.9 | 24.5 | 16.5 | 29.6 |

The fits to the data in Figure 1 show that the distribution of HIV-negative men and women (brown lines) are both skewed to the left with the modal age at death for men being 64 years and for women 74 years while the distribution of HIV-positive men and women (blue lines) are both skew to the right with the modal age at death for men being 37 years and for women being 32 years.

Table 6. Parameters for the fits to the number of men (M) and women (W) who are HIV positive among those suspected of having HIV (S) or whose HIV status is unknown (U).

|  | MS | MU | WS | WU |
| --- | --- | --- | --- | --- |
| Proportion positive | 0.59 | 0.074 | 0.51 | 0.104 |
| Norm | 869 | 1943 | 821 | 1896 |

Table 6 shows that an estimated 59% of the men and 51% of the women who are suspected of having HIV are HIV positive while an estimated 93% of the men and 90% of the women who are thought to be uninfected are HIV negative.

We can now calculate the proportion of false positives and false negatives if we assume that those that that are suspected of being positive are in fact positive and that those whose status is unknown are in fact negative, with the results given in Table 7. For example, if we assume that all those that are suspected of having HIV do in fact have HIV the number of false positive men will be (1−0.59)×869 = 356 which amounts to 356/(869+3672) = 7.8% of those assigned to the HIV-positive category.

Table 7. The proportion of men and women that are misclassified if ‘unknowns’ are treated as negative and ‘suspected’ are treated as positive.

|  | MP | MN | WP | WN |
| --- | --- | --- | --- | --- |
| Prop. misclassified | 0.078 | 0.051 | 0.082 | 0.072 |

Finally, we estimate the effect that this misclassification will have on the odds for being HIV-positive. For the odds in men the numerator will be increased by 7.8% and the denominator will be increased by 5.1% giving an overall increase in the odds ratio of 2.7% (1.078/1.051-1) while for women the increases will be 8.2% and 7.2% giving an overall increase in the odds ratio of 0.9%.

# Appendix 4: Attributable fractions

The attributable fractions for each AIDS-related condition were calculated as follows. Let the number of deaths in the control group (C) and each disease group (D) that are HIV-positive and negative be as indicated in Table 8.

Table 8. The number of deaths in the control group (C) and a given disease group (D) that are HIV positive and HIV-negative.

|  | HIV− | HIV+ |
| --- | --- | --- |
| C | *a* | *b* |
| D | *c* | *d* |

The odds-ratio for being HIV-positive in those with disease D compared to the controls, C, is

$$OR=\frac{ad}{bc}$$

If HIV had no effect on the probability of dying of a particular condition the odds-ration (*OR*) would be 1 and the expected number of HIV-positive deaths from condition D would be

$$\tilde{d}=\frac{bc}{a}$$

The HIV-attributable fraction of deaths in HIV-positive people with condition D is then

$$AF_{\text{D}}^{+}=\frac{d-\frac{bc}{a}}{d}=\frac{bc\left(OR-1\right)}{ad}=\frac{OR-1}{OR}$$

If $P_{D}^{+}$ is the prevalence of HIV in those with condition D the HIV-attributable fraction of deaths in all those, HIV-positive and HIV-negative, with condition D, which we will call the disease attributable fraction, *DAF*, is

$DAF=AF_{D}^{+}P_{D}^{+}$ 5

If *P*D is the prevalence of condition D in the whole sample the population-attributable fraction for HIV due to condition *D* in the whole sample, is

$PAF=DAF\times P_{D}$ 6

Finally, the attributable fraction for all AIDS-related conditions is the sum over Equation 6 for all AIDS-related conditions, *D*.

# Appendix 5. Age-standardization for small numbers

Age-standardization is important as the prevalence of HIV is very high among those less than 50 years of age but much lower among those more than 50 years of age as seen in Figure 5. For certain conditions in which the prevalence of HIV was high, so that the number that were HIV-negative was very small it was not possible to standardize on age. We therefore compared the odds ratios with and without standardization for those conditions for which age-standardization could be done reliably using the data in Table 11 to Table 21 with the result shown in Figure 2. The relationship between the age-standardized odds ratio, *A*, and the crude odds ratio, *C*, is

$$A=\alpha+\beta C$$

where $\alpha=1.019\pm1.261;\beta=0.438\pm0182;\rho=-0.703$ and we us this relationship to estimate the adjusted odds ratios for pulmonary cryptococcus, Kaposi’s sarcoma, *Pneumocystis carinii*, and diseases of the blood and blood forming organs.

Figure 2. Odds ratios standardized for age plotted against crude odds ratios. Blue points: standardized odds ratio measured; Green points: standardized odds ratios for diseases of the blood and blood forming organs and for *Pneumocystis carinii* calculated from the fitted line.

# Appendix 6: Daily mortality

By definition everyone in the sample had died and it is of interest to record the distribution of time between admission and deaths as shown in Figure 3. Mortality after admission was high with a median survival of 4 days and a mean survival of 6.7 days. On the day of admission 10% died, by the next day 27% had died and by the second day after admission 37% had died. By day sixteen 90% had died and by day forty 99% had died.

Figure 3. Deaths on a given number of days after admission on day zero as a proportion of all deaths.

# Appendix 7. Comparison with ANC data for Johannesburg Municipality

The prevalence of HIV in women attending antenatal clinics in the Johannesburg Municipality for the years 2006 to 2009[1-4](#_ENREF_4_1) is significantly less than in the control group used in this study with a peak prevalence at age 30 years of 42% as compared to 68% in the control group. Comparing the two prevalence estimates for those ages where ANC data are available gives an odds ratio for the control group in this study relative to the data from the ante-natal clinic surveys of 3.2 ± 0.7. Figure 4 shows a comparison of the two sets of data after rescaling the ANC data assuming an odds-ratio of 3.2.[1-4](#_ENREF_4_1)

Figure 4. The prevalence of HIV as a function of age in the control group (red) and in the ANC data from Johannesburg Municipality (blue) for the years 2006 to 2009. The ANC data are scaled up assuming an odds-ratio of 3.2.

We then recalculated the *DAF*s and the *PAF*s for each condition, after increasing each of the odds ratios by a factor of 3.2, with the results shown in Figure 5. For cryptococcosis, Kaposi’s sarcoma and *Pneumocystis carinii* the OR is very large so that $AF_{D}^{+}\approx1$and increasing the OR further will have little effect on the *DAF* or *PAF*. For respiratory conditions the OR is low, increasing the OR leads to a significant increase in the *DAF* and since the prevalence is high there will also be a significant increase in the *PAF*. For digestive conditions the OR is again low and increasing the OR leads to a significant increase in the *DAF* but the prevalence of digestive conditions is low so that the *PAF* remains low.

Figure 5. A: The disease-attributable fraction (*DAF*). B: The population-attributable fraction (*PAF*). Black: using the age-specific prevalence in the control group as described in the paper. Red: with the odds-ratios for each condition increased by a factor of 3.2.

# Appendix 8. Prevalence, odds and odds ratios for each disease class

Table 9 to Table 21 give by age, for each category in Table 3, the number of people that were HIV-positive (P+S), HIV-negative (N+U), the prevalence of HIV and the odds for being HIV-positive, with 95% confidence limits.

Table 9. Not coded. Deaths that were not assigned an ICD-10 code.

| Age (yrs) | 15 | 25 | 35 | 45 | 55 | 65 | 75 | 85 |
| --- | --- | --- | --- | --- | --- | --- | --- | --- |
| P+S | 7 | 43 | 112 | 95 | 65 | 18 | 11 | 3 |
| N+U | 4 | 23 | 16 | 47 | 57 | 72 | 54 | 29 |
| Prevalence | 0.636 | 0.652 | 0.875 | 0.669 | 0.533 | 0.200 | 0.169 | 0.094 |
| Lower | 0.308 | 0.524 | 0.805 | 0.585 | 0.440 | 0.123 | 0.088 | 0.020 |
| Upper | 0.891 | 0.765 | 0.927 | 0.746 | 0.624 | 0.298 | 0.283 | 0.250 |
| Odds | 1.750 | 1.870 | 7.000 | 2.021 | 1.140 | 0.250 | 0.204 | 0.103 |
| Lower | 0.445 | 1.102 | 4.127 | 1.411 | 0.787 | 0.140 | 0.096 | 0.020 |
| Upper | 8.151 | 3.250 | 12.667 | 2.932 | 1.657 | 0.424 | 0.394 | 0.334 |
| Ratio | 4.813 | 1.202 | 4.108 | 2.469 | 3.362 | 1.366 | 1.552 | 0.796 |
| Lower | 1.105 | 0.674 | 2.353 | 1.682 | 2.281 | 0.789 | 0.786 | 0.234 |
| Upper | 20.953 | 2.143 | 7.171 | 3.623 | 4.956 | 2.366 | 3.067 | 2.704 |
| Average | 2.299 | 2.299 | 2.299 | 2.299 | 2.299 | 2.299 | 2.299 | 2.299 |
| Upper | 2.794 | 2.794 | 2.794 | 2.794 | 2.794 | 2.794 | 2.794 | 2.794 |
| Lower | 1.891 | 1.891 | 1.891 | 1.891 | 1.891 | 1.891 | 1.891 | 1.891 |

Table 10. Dropped: A02-A07; A32-A39; A49-A87; B01-B18; B37-B43; B46; B50-B58; D01-D46; H52-H70; O90; P20

| Age (yrs) | 15 | 25 | 35 | 45 | 55 | 65 | 75 | 85 |
| --- | --- | --- | --- | --- | --- | --- | --- | --- |
| P+S | 4 | 47 | 79 | 51 | 33 | 10 | 7 | 3 |
| N+U | 6 | 25 | 27 | 41 | 63 | 32 | 39 | 31 |
| Prevalence | 0.400 | 0.653 | 0.745 | 0.554 | 0.344 | 0.238 | 0.152 | 0.088 |
| Lower | 0.122 | 0.531 | 0.651 | 0.447 | 0.250 | 0.121 | 0.063 | 0.019 |
| Upper | 0.738 | 0.761 | 0.825 | 0.658 | 0.448 | 0.395 | 0.289 | 0.237 |
| Odds | 0.667 | 1.880 | 2.926 | 1.244 | 0.524 | 0.313 | 0.179 | 0.097 |
| Lower | 0.138 | 1.134 | 1.869 | 0.808 | 0.333 | 0.137 | 0.068 | 0.019 |
| Upper | 2.811 | 3.187 | 4.713 | 1.925 | 0.810 | 0.652 | 0.406 | 0.310 |
| Ratio | 1.833 | 1.209 | 1.717 | 1.519 | 1.545 | 1.708 | 1.368 | 0.744 |
| Lower | 0.408 | 0.691 | 1.066 | 0.978 | 0.986 | 0.820 | 0.596 | 0.220 |
| Upper | 8.231 | 2.115 | 2.765 | 2.360 | 2.419 | 3.559 | 3.140 | 2.521 |
| Average | 1.489 | 1.489 | 1.489 | 1.489 | 1.489 | 1.489 | 1.489 | 1.489 |
| Upper | 1.842 | 1.842 | 1.842 | 1.842 | 1.842 | 1.842 | 1.842 | 1.842 |
| Lower | 1.204 | 1.204 | 1.204 | 1.204 | 1.204 | 1.204 | 1.204 | 1.204 |

Table 11. Controls: Malignant neo-plasms excluding Kaposi’s sarcoma, Hodgkin’s and Non-Hodgkin’s; disorders involving the immune mechanism; mental; other nervous system; heart and stroke; injury. C02-C45; C59-C80; C83; C90-C96; E03-E88; F01-F30; G10-G99; IU05-I95; Q24-Z91

| Age (yrs) | 15 | 25 | 35 | 45 | 55 | 65 | 75 | 85 |
| --- | --- | --- | --- | --- | --- | --- | --- | --- |
| P+S | 0 | 8 | 126 | 288 | 280 | 214 | 131 | 100 |
| N+U | 1 | 22 | 81 | 169 | 342 | 631 | 716 | 762 |
| Prevalence | 0.000 | 0.267 | 0.609 | 0.630 | 0.450 | 0.253 | 0.155 | 0.116 |
| Lower | 0.000 | 0.123 | 0.539 | 0.584 | 0.411 | 0.224 | 0.131 | 0.095 |
| Upper | 0.010 | 0.459 | 0.676 | 0.675 | 0.490 | 0.284 | 0.181 | 0.139 |
| Odds | 0.000 | 0.364 | 1.556 | 1.704 | 0.819 | 0.339 | 0.183 | 0.131 |
| Lower | 0.000 | 0.140 | 1.167 | 1.404 | 0.697 | 0.289 | 0.151 | 0.105 |
| Upper | 0.010 | 0.848 | 2.083 | 2.073 | 0.962 | 0.397 | 0.221 | 0.162 |

Table 12. Infectious gastroenteritis and colitis, unspecified: A09

| Age (yrs) | 15 | 25 | 35 | 45 | 55 | 65 | 75 | 85 |
| --- | --- | --- | --- | --- | --- | --- | --- | --- |
| P+S | 1 | 62 | 157 | 131 | 60 | 13 | 4 | 1 |
| N+U | 1 | 1 | 13 | 13 | 11 | 12 | 13 | 15 |
| Prevalence | 0.500 | 0.984 | 0.924 | 0.910 | 0.845 | 0.520 | 0.235 | 0.063 |
| Lower | 0.013 | 0.915 | 0.873 | 0.851 | 0.740 | 0.313 | 0.068 | 0.002 |
| Upper | 0.987 | 1.000 | 0.959 | 0.951 | 0.920 | 0.722 | 0.499 | 0.302 |
| Odds | 1.000 | 62.000 | 12.077 | 10.077 | 5.455 | 1.083 | 0.308 | 0.067 |
| Lower | 0.013 | 10.723 | 6.861 | 5.692 | 2.841 | 0.456 | 0.073 | 0.002 |
| Upper | 78.544 | 2485.68 | 23.188 | 19.429 | 11.506 | 2.598 | 0.996 | 0.433 |
| Odds ratio | 2.750 | 39.857 | 7.087 | 12.308 | 16.083 | 5.921 | 2.345 | 0.513 |
| Lower | 0.153 | 5.419 | 3.902 | 6.813 | 8.302 | 2.643 | 0.750 | 0.066 |
| Upper | 49.361 | 293.144 | 12.870 | 22.236 | 31.158 | 13.263 | 7.330 | 3.963 |
| Average | 8.531 | 8.531 | 8.531 | 8.531 | 8.531 | 8.531 | 8.531 | 8.531 |
| Upper | 11.574 | 11.574 | 11.574 | 11.574 | 11.574 | 11.574 | 11.574 | 11.574 |
| Lower | 6.289 | 6.289 | 6.289 | 6.289 | 6.289 | 6.289 | 6.289 | 6.289 |

Table 13. TB: pulmonary A15; A16

| Age (yrs) | 15 | 25 | 35 | 45 | 55 | 65 | 75 | 85 |
| --- | --- | --- | --- | --- | --- | --- | --- | --- |
| P+S | 14 | 206 | 494 | 330 | 174 | 30 | 5 | 0 |
| N+U | 4 | 19 | 24 | 33 | 45 | 32 | 9 | 2 |
| Prevalence | 0.778 | 0.916 | 0.954 | 0.909 | 0.795 | 0.484 | 0.357 |  |
| Lower | 0.524 | 0.871 | 0.932 | 0.875 | 0.735 | 0.355 | 0.128 |  |
| Upper | 0.936 | 0.948 | 0.970 | 0.937 | 0.846 | 0.614 | 0.649 |  |
| Odds | 3.500 | 10.842 | 20.583 | 10.000 | 3.867 | 0.938 | 0.556 |  |
| Lower | 1.099 | 6.768 | 13.672 | 6.981 | 2.772 | 0.550 | 0.146 |  |
| Upper | 14.602 | 18.377 | 32.435 | 14.771 | 5.493 | 1.593 | 1.846 |  |
| Odds ratio | 0.000 | 6.970 | 12.078 | 12.214 | 11.401 | 5.124 | 4.233 |  |
| Lower | 0.000 | 4.035 | 7.689 | 8.260 | 7.934 | 3.011 | 1.391 |  |
| Upper | 0.000 | 12.039 | 18.972 | 18.061 | 16.384 | 8.721 | 12.883 |  |
| Average | 9.620 | 9.620 | 9.620 | 9.620 | 9.620 | 9.620 | 9.620 |  |
| Upper | 11.672 | 11.672 | 11.672 | 11.672 | 11.672 | 11.672 | 11.672 |  |
| Lower | 7.928 | 7.928 | 7.928 | 7.928 | 7.928 | 7.928 | 7.928 |  |

Table 14. TB: extra-pulmonary: A17-A19

| Age (yrs) | 15 | 25 | 35 | 45 | 55 | 65 | 75 | 85 |
| --- | --- | --- | --- | --- | --- | --- | --- | --- |
| P+S | 4 | 141 | 368 | 244 | 88 | 17 | 3 | 2 |
| N+U | 1 | 10 | 20 | 23 | 17 | 6 | 3 | 2 |
| Prevalence | 0.800 | 0.934 | 0.948 | 0.914 | 0.838 | 0.739 | 0.500 | 0.500 |
| Lower | 0.284 | 0.882 | 0.922 | 0.874 | 0.753 | 0.516 | 0.118 | 0.068 |
| Upper | 0.995 | 0.968 | 0.968 | 0.945 | 0.903 | 0.898 | 0.882 | 0.932 |
| Odds | 4.000 | 14.100 | 18.400 | 10.609 | 5.176 | 2.833 | 1.000 | 1.000 |
| Lower | 0.396 | 7.445 | 11.741 | 6.908 | 3.056 | 1.066 | 0.134 | 0.072 |
| Upper | 196.973 | 30.044 | 30.480 | 17.053 | 9.284 | 8.776 | 7.466 | 13.796 |
| Odds ratio | 11.000 | 9.064 | 10.797 | 12.958 | 15.263 | 15.486 | 7.620 | 7.692 |
| Lower | 1.064 | 4.503 | 6.625 | 8.215 | 8.878 | 5.994 | 1.517 | 1.061 |
| Upper | 113.736 | 18.244 | 17.597 | 20.439 | 26.242 | 40.009 | 38.269 | 55.779 |
| Average | 12.112 | 12.112 | 12.112 | 12.112 | 12.112 | 12.112 | 12.112 | 12.112 |
| Upper | 15.506 | 15.506 | 15.506 | 15.506 | 15.506 | 15.506 | 15.506 | 15.506 |
| Lower | 9.461 | 9.461 | 9.461 | 9.461 | 9.461 | 9.461 | 9.461 | 9.461 |

Table 15. Other sepsis, Infectious and parasitic: A41

| Age (yrs) | 15 | 25 | 35 | 45 | 55 | 65 | 75 | 85 |
| --- | --- | --- | --- | --- | --- | --- | --- | --- |
| P+S | 6 | 84 | 184 | 154 | 73 | 26 | 9 | 4 |
| N+U | 3 | 17 | 25 | 49 | 47 | 81 | 69 | 46 |
| Prevalence | 0.667 | 0.832 | 0.880 | 0.759 | 0.608 | 0.243 | 0.115 | 0.080 |
| Lower | 0.299 | 0.744 | 0.829 | 0.694 | 0.515 | 0.165 | 0.054 | 0.022 |
| Upper | 0.925 | 0.899 | 0.921 | 0.816 | 0.696 | 0.335 | 0.208 | 0.192 |
| Odds | 2.000 | 4.941 | 7.360 | 3.143 | 1.553 | 0.321 | 0.130 | 0.087 |
| Lower | 0.427 | 2.910 | 4.831 | 2.265 | 1.062 | 0.198 | 0.057 | 0.023 |
| Upper | 12.359 | 8.880 | 11.670 | 4.428 | 2.291 | 0.505 | 0.262 | 0.238 |
| Odds ratio | 5.500 | 3.176 | 4.319 | 3.839 | 4.580 | 1.754 | 0.994 | 0.669 |
| Lower | 1.105 | 1.759 | 2.729 | 2.683 | 3.076 | 1.086 | 0.481 | 0.231 |
| Upper | 27.375 | 5.738 | 6.834 | 5.492 | 6.818 | 2.834 | 2.053 | 1.934 |
| Average | 3.100 | 3.100 | 3.100 | 3.100 | 3.100 | 3.100 | 3.100 | 3.100 |
| Upper | 3.729 | 3.729 | 3.729 | 3.729 | 3.729 | 3.729 | 3.729 | 3.729 |
| Lower | 2.576 | 2.576 | 2.576 | 2.576 | 2.576 | 2.576 | 2.576 | 2.576 |

Table 16. Hodgkin’s and non-Hodgkin’s lymphoma: C81; C85

| Age (yrs) | 15 | 25 | 35 | 45 | 55 | 65 | 75 | 85 |
| --- | --- | --- | --- | --- | --- | --- | --- | --- |
| P+S | 0 | 7 | 34 | 21 | 6 | 2 | 0 | 1 |
| N+U | 1 | 8 | 2 | 6 | 3 | 3 | 1 | 0 |
| Prevalence | 0.000 | 0.467 | 0.944 | 0.778 | 0.667 | 0.400 | 0.000 |  |
| Lower | 0.000 | 0.213 | 0.813 | 0.577 | 0.299 | 0.053 | 0.000 |  |
| Upper | 0.010 | 0.734 | 0.993 | 0.914 | 0.925 | 0.853 | 0.200 |  |
| Odds | 0.000 | 0.875 | 17.000 | 3.500 | 2.000 | 0.667 | 0.000 |  |
| Lower | 0.000 | 0.270 | 4.358 | 1.366 | 0.427 | 0.056 | 0.000 |  |
| Upper | 0.010 | 2.761 | 146.045 | 10.599 | 12.359 | 5.820 | 0.250 |  |
| Odds ratio | 0.000 | 0.563 | 9.976 | 4.275 | 5.897 | 3.644 | 0.000 |  |
| Lower | 0.000 | 0.196 | 2.367 | 1.702 | 1.462 | 0.603 | 0.000 |  |
| Upper | 0.000 | 1.611 | 42.050 | 10.737 | 23.785 | 22.019 | 0.000 |  |
| Average | 2.907 | 2.907 | 2.907 | 2.907 | 2.907 | 2.907 | 0.000 |  |
| Upper | 5.005 | 5.005 | 5.005 | 5.005 | 5.005 | 5.005 | 0.000 |  |
| Lower | 1.689 | 1.689 | 1.689 | 1.689 | 1.689 | 1.689 | 0.000 |  |

Table 17. Meningitis: G00-G03

| Age (yrs) | 15 | 25 | 35 | 45 | 55 | 65 | 75 | 85 |
| --- | --- | --- | --- | --- | --- | --- | --- | --- |
| P+S | 5 | 79 | 229 | 154 | 50 | 5 | 4 | 0 |
| N+U | 9 | 15 | 38 | 44 | 16 | 20 | 12 | 4 |
| Prevalence | 0.357 | 0.840 | 0.858 | 0.778 | 0.758 | 0.200 | 0.250 |  |
| Lower | 0.128 | 0.751 | 0.810 | 0.713 | 0.636 | 0.068 | 0.073 |  |
| Upper | 0.649 | 0.908 | 0.897 | 0.834 | 0.855 | 0.407 | 0.524 |  |
| Odds ratio | 0.556 | 5.267 | 6.026 | 3.500 | 3.125 | 0.250 | 0.333 |  |
| Lower | 0.146 | 3.010 | 4.259 | 2.490 | 1.751 | 0.073 | 0.078 |  |
| Upper | 1.850 | 9.852 | 8.734 | 5.011 | 5.881 | 0.687 | 1.100 |  |
| Ratio | 1.528 | 3.386 | 3.536 | 4.275 | 9.214 | 1.366 | 2.540 |  |
| Lower | 0.494 | 1.824 | 2.389 | 2.952 | 5.139 | 0.504 | 0.804 |  |
| Upper | 4.721 | 6.285 | 5.235 | 6.192 | 16.522 | 3.705 | 8.027 |  |
| Average | 3.913 | 3.913 | 3.913 | 3.913 | 3.913 | 3.913 | 3.913 |  |
| Upper | 4.845 | 4.845 | 4.845 | 4.845 | 4.845 | 4.845 | 4.845 |  |
| Lower | 3.160 | 3.160 | 3.160 | 3.160 | 3.160 | 3.160 | 3.160 |  |

Table 18. Respiratory, Pneumonia, COPD; lower respiratory: J01-J99

| Age (yrs) | 15 | 25 | 35 | 45 | 55 | 65 | 75 | 85 |
| --- | --- | --- | --- | --- | --- | --- | --- | --- |
| P+S | 18 | 286 | 648 | 545 | 266 | 73 | 42 | 13 |
| N+U | 10 | 65 | 91 | 123 | 217 | 211 | 142 | 90 |
| Prevalence | 0.643 | 0.815 | 0.877 | 0.816 | 0.551 | 0.257 | 0.228 | 0.126 |
| Lower | 0.441 | 0.770 | 0.851 | 0.784 | 0.505 | 0.207 | 0.170 | 0.069 |
| Upper | 0.814 | 0.854 | 0.900 | 0.845 | 0.596 | 0.312 | 0.296 | 0.206 |
| Odds ratio | 1.800 | 4.400 | 7.121 | 4.431 | 1.226 | 0.346 | 0.296 | 0.144 |
| Lower | 0.788 | 3.351 | 5.710 | 3.637 | 1.021 | 0.261 | 0.204 | 0.074 |
| Upper | 4.365 | 5.853 | 8.969 | 5.433 | 1.473 | 0.453 | 0.420 | 0.260 |
| Ratio | 4.950 | 2.829 | 4.179 | 5.412 | 3.614 | 1.891 | 2.254 | 1.111 |
| Lower | 1.617 | 1.919 | 3.126 | 4.209 | 2.852 | 1.366 | 1.524 | 0.603 |
| Upper | 15.157 | 4.169 | 5.585 | 6.959 | 4.581 | 2.617 | 3.334 | 2.046 |
| Average | 3.322 | 3.322 | 3.322 | 3.322 | 3.322 | 3.322 | 3.322 | 3.322 |
| Upper | 3.738 | 3.738 | 3.738 | 3.738 | 3.738 | 3.738 | 3.738 | 3.738 |
| Lower | 2.952 | 2.952 | 2.952 | 2.952 | 2.952 | 2.952 | 2.952 | 2.952 |

Table 19. Digestive, Liver: K25-K95

| Age (yrs) | 15 | 25 | 35 | 45 | 55 | 65 | 75 | 85 |
| --- | --- | --- | --- | --- | --- | --- | --- | --- |
| P+S | 1 | 15 | 42 | 32 | 15 | 6 | 2 | 2 |
| N+U | 3 | 8 | 9 | 30 | 31 | 24 | 13 | 5 |
| Prevalence | 0.250 | 0.652 | 0.824 | 0.516 | 0.326 | 0.200 | 0.133 | 0.286 |
| Lower | 0.006 | 0.427 | 0.691 | 0.386 | 0.195 | 0.077 | 0.017 | 0.037 |
| Upper | 0.806 | 0.836 | 0.916 | 0.645 | 0.480 | 0.386 | 0.405 | 0.710 |
| Odds | 0.333 | 1.875 | 4.667 | 1.067 | 0.484 | 0.250 | 0.154 | 0.400 |
| Lower | 0.006 | 0.746 | 2.239 | 0.628 | 0.243 | 0.084 | 0.017 | 0.038 |
| Upper | 4.152 | 5.106 | 10.903 | 1.817 | 0.924 | 0.628 | 0.680 | 2.443 |
| Odds ratio | 0.917 | 1.205 | 2.738 | 1.303 | 1.427 | 1.366 | 1.172 | 3.077 |
| Lower | 0.083 | 0.489 | 1.301 | 0.773 | 0.756 | 0.548 | 0.261 | 0.591 |
| Upper | 10.140 | 2.972 | 5.766 | 2.197 | 2.694 | 3.408 | 5.256 | 16.028 |
| Average | 1.497 | 1.497 | 1.497 | 1.497 | 1.497 | 1.497 | 1.497 | 1.497 |
| Upper | 2.037 | 2.037 | 2.037 | 2.037 | 2.037 | 2.037 | 2.037 | 2.037 |
| Lower | 1.100 | 1.100 | 1.100 | 1.100 | 1.100 | 1.100 | 1.100 | 1.100 |

Table 20. Skin and bone: L03-L95; M01-M99

| Age (yrs) | 15 | 25 | 35 | 45 | 55 | 65 | 75 | 85 |
| --- | --- | --- | --- | --- | --- | --- | --- | --- |
| P+S | 2 | 8 | 20 | 17 | 12 | 7 | 0 | 2 |
| N+U | 1 | 4 | 9 | 12 | 16 | 25 | 10 | 2 |
| Prevalence | 0.667 | 0.667 | 0.690 | 0.586 | 0.429 | 0.219 | 0.000 | 0.500 |
| Lower | 0.094 | 0.350 | 0.491 | 0.390 | 0.244 | 0.093 | 0.100 | 0.068 |
| Upper | 0.992 | 0.901 | 0.847 | 0.765 | 0.628 | 0.400 | 0.308 | 0.932 |
| Odds | 2.000 | 2.000 | 2.222 | 1.417 | 0.750 | 0.280 | 0.000 | 1.000 |
| Lower | 0.104 | 0.538 | 0.966 | 0.639 | 0.323 | 0.102 | 0.111 | 0.073 |
| Upper | 116.768 | 9.067 | 5.534 | 3.256 | 1.690 | 0.666 | 0.446 | 13.787 |
| Odds ratio | 1.286 | 1.174 | 2.714 | 4.177 | 4.099 | 2.134 | 0.000 | 9.143 |
| Lower | 0.115 | 0.348 | 1.217 | 1.963 | 1.896 | 0.904 | #NUM! | 1.277 |
| Upper | 14.411 | 3.956 | 6.055 | 8.888 | 8.865 | 5.035 | #NUM! | 65.484 |
| Average | 2.880 | 2.880 | 2.880 | 2.880 | 2.880 | 2.880 | 2.880 | 2.880 |
| Upper | 4.182 | 4.182 | 4.182 | 4.182 | 4.182 | 4.182 | 4.182 | 4.182 |
| Lower | 1.984 | 1.984 | 1.984 | 1.984 | 1.984 | 1.984 | 1.984 | 1.984 |

Table 21. Genito-urinary infections: N00-N92.

| Age (yrs) | 15 | 25 | 35 | 45 | 55 | 65 | 75 | 85 |
| --- | --- | --- | --- | --- | --- | --- | --- | --- |
| P+S | 2 | 57 | 130 | 153 | 70 | 26 | 17 | 9 |
| N+U | 7 | 20 | 28 | 51 | 90 | 100 | 61 | 45 |
| Prevalence | 0.222 | 0.740 | 0.823 | 0.750 | 0.438 | 0.206 | 0.218 | 0.167 |
| Lower | 0.028 | 0.628 | 0.754 | 0.685 | 0.359 | 0.139 | 0.132 | 0.079 |
| Upper | 0.600 | 0.834 | 0.879 | 0.808 | 0.518 | 0.288 | 0.326 | 0.293 |
| Odds ratio | 0.286 | 2.850 | 4.643 | 3.000 | 0.778 | 0.260 | 0.279 | 0.200 |
| Lower | 0.029 | 1.686 | 3.068 | 2.172 | 0.561 | 0.162 | 0.153 | 0.086 |
| Upper | 1.501 | 5.008 | 7.257 | 4.204 | 1.075 | 0.404 | 0.483 | 0.414 |
| Ratio | 0.184 | 1.672 | 5.671 | 8.846 | 4.251 | 1.981 | 2.144 | 1.829 |
| Lower | 0.037 | 0.971 | 3.660 | 6.216 | 2.955 | 1.238 | 1.214 | 0.873 |
| Upper | 0.906 | 2.880 | 8.786 | 12.588 | 6.116 | 3.170 | 3.787 | 3.831 |
| Average | 4.185 | 4.185 | 4.185 | 4.185 | 4.185 | 4.185 | 4.185 | 4.185 |
| Upper | 5.037 | 5.037 | 5.037 | 5.037 | 5.037 | 5.037 | 5.037 | 5.037 |
| Lower | 3.477 | 3.477 | 3.477 | 3.477 | 3.477 | 3.477 | 3.477 | 3.477 |

The data in Table 9 to Table 21 are plotted against age with the overall estimate of the OR and 95% confidence limits. The confidence limits on the average values are calculated using only the binomial sampling errors. For genitor-urinary conditions there is further variability over and above the sampling errors.

TB: pulmonary

Figure 6. Odds-ratios by age for each of the conditions noted on the figure with the average values and 95% confidence limits for the points and for the average value.

TB: extra-pulmonary

Lymphoma

# References

1. Anonymous 2007. *National HIV and syphilis antenatal sero-prevalence survey in South Africa 2006.* Pretoria: Directorate: Health Systems Research, Research Coordination and Epidemiology.

2. Anonymous 2008. *National HIV and syphilis antenatal sero-prevalence survey in South Africa 2007.* Pretoria: Directorate: Health Systems Research, Research Coordination and Epidemiology.

3. Anonymous 2009. *National Antenatal Sentinel HIV and Syphilis Prevalence Survey, South Africa 2008.* Pretoria: Department of Health, South Africa.

4. Anonymous 2010. *National Antenatal Sentinel HIV and Syphilis Prevalence Survey in South Africa, 2009.* Department of Health, South Africa.
